# Novel pituitary actions of GnRH in teleost: The link between reproduction and feeding regulation

**DOI:** 10.3389/fendo.2022.982297

**Published:** 2022-10-11

**Authors:** Wei Li, Ruixin Du, Chuanhui Xia, Huiying Zhang, Yunyi Xie, Xiaowen Gao, Yu Ouyang, Zhan Yin, Guangfu Hu

**Affiliations:** ^1^ Hubei Province Engineering Laboratory for Pond Aquaculture, College of Fisheries, Huazhong Agricultural University, Wuhan, China; ^2^ State Key Laboratory of Freshwater Ecology and Biotechnology, Institute of Hydrobiology, Chinese Academy of Sciences, Wuhan, China

**Keywords:** GnRH, reproduction, feeding, grass carp, pituitary

## Abstract

Gonadotropin-releasing hormone (GnRH), as a vital hypothalamic neuropeptide, was a key regulator for pituitary luteinizing hormone (LH) and follicle-stimulating hormone (FSH) in the vertebrate. However, little is known about the other pituitary actions of GnRH in teleost. In the present study, two GnRH variants (namely, GnRH2 and GnRH3) and four GnRH receptors (namely, GnRHR1, GnRHR2, GnRHR3, and GnRHR4) had been isolated from grass carp. Tissue distribution displayed that GnRHR4 was more highly detected in the pituitary than the other three GnRHRs. Interestingly, ligand–receptor selectivity showed that GnRHR4 displayed a similar and high binding affinity for grass carp GnRH2 and GnRH3. Using primary culture grass carp pituitary cells as model, we found that both GnRH2 and GnRH3 could not only significantly induce pituitary reproductive hormone gene (GtHα, LHβ, FSHβ, INHBa, secretogranin-2) mRNA expression mediated by AC/PKA, PLC/IP_3_/PKC, and Ca^2+^/CaM/CaMK-II pathways but also reduce dopamine receptor 2 (DRD2) mRNA expression *via* the Ca^2+^/CaM/CaMK-II pathway. Interestingly, GnRH2 and GnRH3 could also stimulate anorexigenic peptide (POMCb, CART2, UTS1, NMBa, and NMBb) mRNA expression *via* AC/PKA, PLC/IP_3_/PKC, and Ca^2+^/CaM/CaMK-II pathways in grass carp pituitary cells. In addition, food intake could significantly induce brain GnRH2 mRNA expression. These results indicated that GnRH should be the coupling factor to integrate the feeding metabolism and reproduction in teleost.

## Introduction

As an extremely important hypothalamus neuroendocrine peptide, gonadotropin releasing hormone 1 (GnRH1) was initially isolated from the mammalian hypothalamus (1, 2). Subsequently, GnRH2 had been firstly found in chicken so that this variant was also named as chicken GnRH (cGnRH) (3). In addition, the third GnRH variant (GnRH3) was an ubiquitous and unique subtype existing in fish (3), which was primitively discovered in salmon so that it was named as salmon GnRH (sGnRH) (4). Multiple GnRH variants (two or three forms) are present in all teleosts (5), but the receptor selectivity and functions of different GnRH variants are still unclear in teleost. Similar to mammals, GnRH could also participate in the reproductive regulation in the teleost, including spawning activity (6) and oocyte development (7). A recent study further found that GnRH3 could regulate primordial germ cell (PGC) proliferation and sex differentiation in zebrafish (8). In addition, GnRH2 knockout zebrafish females display decreased oocyte quality (9). In teleost, several studies reported that the two GnRH variants (namely, GnRH2 and GnRH3) could stimulate luteinizing hormone (LH) and follicle-stimulating hormone (FSH) secretion in the pituitary (5, 10). However, besides LH and FSH, could GnRH regulate other reproductive genes in the pituitary?

As we know, energy metabolism was associated with reproductive behavior in vertebrates (11), and adequate energy reserve was essential for breeding (12). Previous studies suggested that several hypothalamus neuropeptides could regulate both reproduction and feeding in teleost (13). Our recent study also found that neurokinin B (NKB) could regulate not only reproduction but also feeding in grass carp (14). In addition, prolactin-releasing peptide (PRRP), a typical anorexigenic peptide in hypothalamus, could significantly induce LH secretion and synthesis in grass carp pituitary (15). Similarly, GnRH, the typical reproductive peptide in hypothalamus, could also regulate food intake in mammals (16) and teleosts (17). Recent studies further confirmed that knockout of gnrh2 in zebrafish could increase food intake (9). However, little is known about the regulatory mechanism of GnRH in feeding regulation.

In this study, grass carp were used as a model to examine the pituitary actions of GnRH in reproduction and feeding. Firstly, two GnRHs and four GnRH receptors (GnRHRs) were isolated from grass carp, and the tissue distribution of these genes was examined by using specific primers. Then, ligand–receptor selectivity was performed by the established pGL3-nuclear factor of activated T cell (NFAT)-RE-luciferase reporters in HEK293-T cells. Besides, using primary culture grass carp pituitary cells as a model, direct pituitary actions of GnRH3 were examined by RNA-seq technique. Afterward, we further confirmed that GnRHs could significantly regulate five pituitary reproductive hormone genes (GtHα, LHβ, FSHβ, INHBa, secretogranin-2) and five anorexigenic peptides (POMCb, CART2, UTS1, NMBa, and NMBb) in grass carp pituitary cells. Finally, we further examined the signal pathways of GnRH-regulated reproductive and feeding genes in the pituitary. Our findings demonstrated the functional roles of GnRH in the regulation of reproduction and feeding in the teleost.

## Materials and methods

### Animals and chemicals

In the present study, 2-year-old grass carp (*Ctenopharyngodon idellus*) with a body weight of 1.5–2.5 kg were acquired from local markets and maintained in 250-l aquaria under a 12-h light, 12-h dark photoperiod at 20°C. Because sexual dimorphism was not apparent in these fish, grass carps of mixed sexes were used for pituitary cell preparation according to the protocol approved by the committee for animal use at Huazhong Agricultural University. Grass carp GnRH2 (QHWSHGWYPG-NH_2_), GnRH3 (QHWSYGWLPG-NH_2_), and human GnRH1 (QHWSYGLRPG-NH_2_) were synthesized by GenScript (Piscataway, NJ) and dissolved in double-distilled water at 1 mM which were sub-packaged and stored at lower than -80°C. The full-length open reading frame (ORF) of grass carp GnRH receptors (GnRHR1, GnRHR2, GnRHR3, GnRHR4) were cloned and then inserted into pcDNA3.1(+) vector (Invitrogen) used for transfection, while human GnRHR (GenBank No: L07949.1) was synthesized by BT Lab (Wuhan, China). All the signal pathway inhibitors, such as H89, MDL12330A, U73122, GF109203X, 2-APB, nifedipine, KN62, and calmidazolium (CMZ), were purchased from Calbiochem (San Diego, CA) (for details, please refer to **Supplementary Table S1**) and dissolved using dimethyl sulfoxide (DMSO) at a concentration of 10 mM. Once being used in *in vitro* tests, these drugs were diluted to working concentration by testing medium beforehand.

### Molecular cloning and tissue distribution of grass carp Gnrhs and Gnrhrs

Total RNA was extracted from grass carp pituitary and hypothalamus and reverse transcribed into cDNA with Hifair™ III 1st Strand cDNA Synthesis Kit (Yeasen, Shanghai, China). The full-length ORF regions of grass carp GnRHR1, GnRHR2, GnRHR3, and GnRHR4 were cloned using specific primers designed based on grass carp genomes, respectively (for the conditions of primers, please refer to **Supplementary Table S2**). The sequence alignment based on the corresponding cDNA or mature peptide sequences which were reported in other species was conducted with BioEdit 7.2, and phylogenetic analysis of target sequences was conducted with MEGA7.0 and ClustalX 2.1 using the neighbor-joining method. The three-dimensional protein models of grass carp GnRH2, GnRH3, GnRHR1, GnRHR2, GnRHR3, and GnRHR4 were predicted and constructed by using SWISS-MODEL and I-TASSER based on the deduced amino acid sequence. For tissue distribution analysis, the total RNA of various brain subregions and several selected peripheral tissues were isolated and reverse transcribed to cDNA to detect the transcript level using primers specific for gene targets by real-time PCR (RT-PCR), respectively (for the conditions of primers, please refer to **Supplementary Table S3**). In these studies, RT-PCR for β-actin was performed as an internal control.

### Transfection and luciferase reporter assay

According to our previous study, the pGL3-NFAT-RE-luciferase reporter system was used to verify the ligand–receptor selectivity of the newly cloned GnRHRs in HEK-293T cells (18). Briefly, the ORFs of grass carp GnRHR1, GnRHR2, GnRHR3, GnRHR4, and hGnRHR were isolated and subcloned into eukaryotic expression vector pcDNA3.1(+) to generate corresponding expression vectors. For transient transfection experiments, HEK-293T cell lines were seeded at a density of 0.05 × 10^6^ cells/0.5 ml/well in 24-well plates. After overnight incubation for recovery, transfection was carried out in 400 μf OPTI-MEM for 6 h with 200 ng NFAT-Luc reporter or CRE-Luc reporter, 10 ng pTK-RL, 20 ng pEGFP-N1, 10 ng pcDNA3.1(+)-GnRHR, and 0.99 μl Lipofectamine 3000 (Thermo Fisher). pTK-RL (the Renilla luciferase-expressing reporter) and GFP-expressing vector pEGFP-N1 were both used as the internal control. Parallel transfection with the blank vector pcDNA3.1(+) without GnRHR insert was used as the negative control. After transfection, the cells were allowed to incubate for 18–24 h at 37°C in Dulbecco’s modified Eagle medium (DMEM) supplemented without fetal bovine serum (FBS, Gibco) before drug treatment. Based on our validation, the duration of drug treatment has been optimized for 24 h for luciferin expression. After a 24-h drug treatment, the cells were washed with ice-cold PBS and dissolved in passive lysis buffer (Yeasen, Shanghai, China). The prepared cellular lysate was then used for the measurement of firefly luciferase activities using Luciferase Assay Reagent (Yeasen, Shanghai, China) by a dual luciferase reporter system. Furthermore, transfection experiments were performed in quadruplicate with cells cultured in separate wells.

### RNA-seq and bioinformatics

The grass carp pituitaries were obtained and dispersed by the trypsin/DNase II/EDTA digestion method (19). Grass carp pituitary cells were seeded in 24-well plates and initially cultured in a plating medium at the density of 2.5 × 10^6^ cells/0.8 ml per well under the condition of 28°C with 5% CO_2_. After adding 5% FBS to each well and incubating for 18 h, GnRH3 (final concentration of 1 μM) was used to incubate the pituitary cells for another 24 h. Then, total RNA was extracted from each well by TRIzol reagent (Yeasen) and DNase II was used to eliminate the interference of genomic DNA. The concentration and purity of each RNA sample were detected by a NanoDrop 2000 spectrophotometer, while the quality of RNA was identified on an Agilent 2100 Bioanalyzer using the RNA 6000 Nano Kit (Agilent Technologies, Santa Clara, CA, USA). After that, the RNA (RIN >8.0) including the control group and GnRH-treated group (both three replicates) were sent to Majorbio Genome Center (Shanghai, China) for subsequent library preparation and sequencing on HiSeq 4000 (Illumina). In this study, a read depth of 0.6 billion 150-bp single-end reads was used and about 90% of reads were mapped to the genome. Gene expression levels were assigned individual values by being normalized to the number of transcripts per kilobase of exon model per million (TPM). The fold changes (FC) were calculated using RSEM software v 1.2.7 (20), and different gene expressions (DEGs) were analyzed by using the R Bioconductor package. The P-value indicated the credibility of each differential gene expression and was corrected by the false discovery rate (FDR) (21). We set up conditions of TPM >5, FDR <0.05, and FC >1.5 to select satisfactory DEGs. Finally, both Gene Ontology (GO) and Kyoto Encyclopedia of Genes and Genomes (KEGG) enrichment analyses were performed using Goatools software (22).

### Quantitative real-time PCR in pituitary cells

The preparation of grass carp pituitary cells was performed as mentioned above. After drug treatment, the total RNA of these cells was isolated by TRIzol reagent and reverse transcribed into cDNA with Hifair™ III 1st Strand cDNA Synthesis Kit. The transcript levels of several anorectic peptides (POMCb, CART2, UTS1, NMBa, NMBb, and Lepr) and reproductive genes (GtHα, LHβ, FSHβ, INHBa, SgII, DRD2) were detected by using an ABI 7500 quantitative real-time PCR (qRT-PCR) system (Biosystems, USA) (information of primers for target genes is listed in **Supplementary Table S3**). In this process, a serial gradient dilution of plasmid DNA of these genes was used as a standard for data calibration. The conditions of qRT-PCR were set to 10 min, 95°C, for pre-degeneration; 15 s, 95°C, for degeneration; 30 s, 55°C–60°C, for annealing; 30 s, 72°C, for extending; and 20 s, 82°C, for signal detection with 40 cycles. Finally, the melt curve obtained through each test was used to verify and check the reliability and specificity of the corresponding qRT-PCR.

### Postprandial changes in GnRH expression

To further confirm the potential functional role of GnRH on feeding, we detected the GnRH2 and GnRH3 mRNA expression in grass carp brain after feeding. Grass carp were temporarily raised in a well-aerated 250-l tank and fed one meal per day for at least 7 days at fixed times (9:00 a.m.). The grass carp were divided into equal portions as feed group and unfed group (as a control group). On the experiment day, the food supply point (9:00 a.m.) was considered as 0 h. Therefore, the brains were harvested at 0, 1, 3, and 6 h after food administration from these two groups, respectively. Then, the total RNA was extracted by TRIzol method and transcribed into cDNA to detect the GnRH2 and GnRH3 mRNA expression by the qRT-PCR system.

### Data transformation and statistical analysis

The transcript level was detected using qRT-PCR by ABI 7500 software, while the data calibration of each reaction was performed through standard curves with the dynamic range of 10^5^ and correlation coefficient >0.95. The transcript level of β-actin was used as an internal control, and target gene mRNA expression was normalized and calculated as a percentage of the mean value (as “% Ctrl”). Based on merging four to eight replicates (as mean ± SEM), the data were analyzed with a one-way ANOVA to differentiate the significant differences from other treatment experiments. The SPSS Statistics 26.0 software was used to do a Dunnett’s *post-hoc* test. Finally, *p* < 0.05 (“*”) or *p* < 0.01 (“**”) was used to present significant differences among each group. The different letters represent a significant difference at *p* < 0.05 between groups.

## Results

### Molecular cloning and sequence analysis of GnRHs and GnRHRs in grass carp

**Figure 1:**
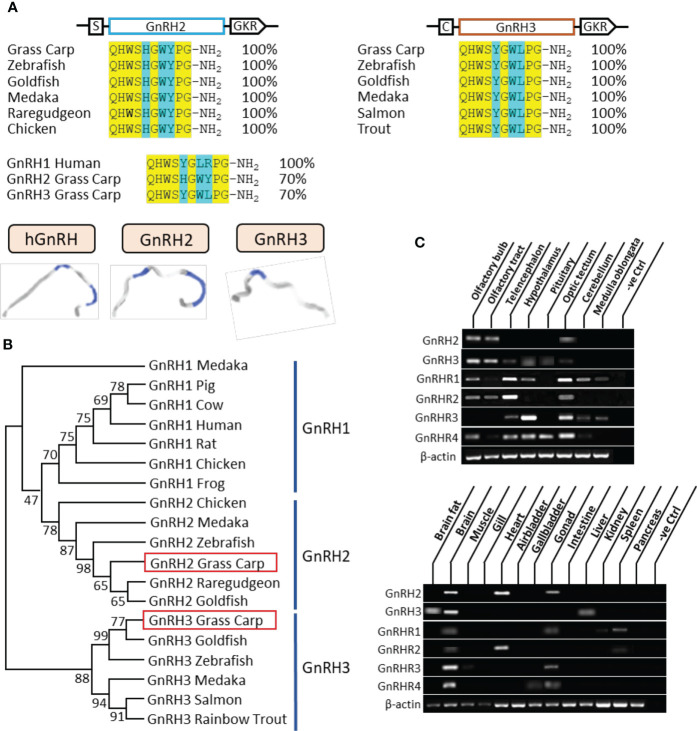
Sequence analysis and tissue distribution of grass carp GnRHs/GnRHRs. **(A)** The mature peptide sequence alignment of GnRHs. The conserved amino acid sequences are processed into a yellow background, whereas the different amino acid residues compared with human GnRH1, grass carp GnRH2, and GnRH3 are marked in blue background. **(B)** Phylogenetic analysis of GnRHs from mammal or non-mammal vertebrates are generated with the neighbor-joining method (MEGA 6.0); grass carp GnRH2 and GnRH3 are highlighted into the red frame. **(C)** Tissue distribution of GnRHs and GnRHRs was detected in grass carp peripheral tissues (on the bottom) and various brain subregions (on the top). Total RNA was extracted, reverse transcribed, and underwent RT-PCR using specific primers; the results have been intercepted and spliced according to corresponding PCR product size. Besides, the transcript level of β-actin was considered as an internal control.

In grass carp, the full lengths of GnRH2 and GnRH3 were cloned by using specific primers. According to sequence alignment, we found that the ORF of GnRH2 contained 261 bp in size as well as an encoded 86-amino acid protein precursor, while GnRH3 possessed 285 bp in size and an encoded 94-amino acid protein precursor (**Supplementary Figure S1**). Besides, GnRH2 and GnRH3 encoded one 10-aa mature peptide (QHWSHGWYPG-NH2 and QHWSYGWLPG-NH2, respectively), which were both with the common motif (PG-NH2) in the C terminus (**Figure 1A**). At the protein level, we compared the mature peptides of GnRH in different species; the results indicated that grass carp GnRH2 showed 100% identity to all the contrastive counterparts including zebrafish, goldfish, medaka, and chicken. Similar to GnRH2, grass carp GnRH3 revealed 100% identity to the counterparts in teleost (**Figure 1A**). The three-dimensional protein structures for human GnRH1, grass carp GnRH2, and grass carp GnRH3 were predicted by using I-TASSER (**Figure 1A**). The phylogenetic analysis revealed that the two different subtypes of GnRHs were clustered into separate branches, and GnRH2 showed a closer relationship to GnRH1 compared with GnRH3 which was a unique isoform for teleost (**Figure 1B**). GnRHR1, GnRHR2, GnRHR3, and GnRHR4 had been cloned from grass carp pituitary, which encoded 381, 414, 373, and 406 aa proteins, respectively. The amino acid sequence of the four receptors, as members of the GPCR group, could be structured into seven transmembrane domains (TMD 1 to 7) with three extracellular loops and three intracellular loops, together with an endocellular C-terminal and an extracellular N-terminal tail (**Supplementary Figures S2-5**). Similarly, phylogenetic analysis showed that grass carp GnRHR1 and GnRHR3 were clustered in the same brand, which was close to mammalian GnRHR. Besides, GnRHR2 and GnRHR4 were clustered into a distinct brand (**Supplementary Figure S6**).

The tissue distribution showed that GnRH2 was mainly distributed in the brain, heart, and gonad, whereas GnRH3 primarily existed in the brain and liver (**Figure 1C**). At the brain level, GnRH2 was highly detected in the olfactory bulb, olfactory tract, and optic tectum. However, high transcript levels of GnRH3 were detected in the olfactory bulb, olfactory tract, hypothalamus, and pituitary (**Figure 1C**). The four GnRHRs were mainly detected in the brain and gonad (**Figure 1C**). In various brain subregions, the transcript signals of all the four receptors were detected in the telencephalon, optic tectum, and hypothalamus (**Figure 1C**).

### Ligand–receptor selectivity of GnRHs for GnRHRs in HEK-293T cells

**Figure 2:**
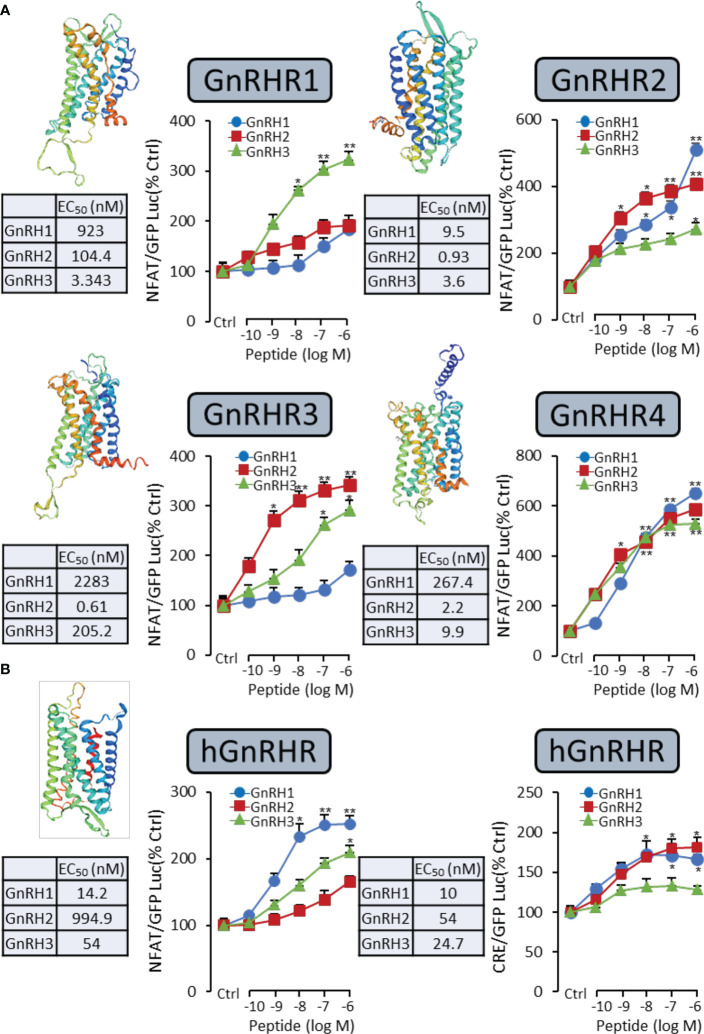
Functional analysis of grass carp GnRHRs and human GnRHR in HEK-293T cells. To examine the ligand–receptor selectivity of the GnRHs for GnRHRs, the NFAT-Luc or CRE-Luc reporter system was used in HEK-293T cells which were treated with various concentrations of GnRHs for 24 h, to detect the luciferase activity of GnRHs for grass carp GnRHR1, GnRHR2, GnRHR3, GnRHR4 **(A)**, and hGnRHR **(B)**. Each point was determined in quadruplicate, and data presented were expressed as mean ± SEM. *p* < 0.05 (“*”) or *p* < 0.01 (“**”) was used to present significant differences among each group.

It had been reported that the GnRH-induced rapid increase in intracellular calcium was essential for gonadotropin secretion (17). Therefore, a pGL3-NFAT-RE-luciferase reporter system, which could monitor the changes in intracellular calcium concentration, was used in the present study. As shown in **Figure 2A** for grass carp GnRHR, the GnRH variants (GnRH1, GnRH2, and GnRH3) were all effective in stimulating luciferase activity expression in a dose-dependent manner *via* the Ca^2+^ pathway. GnRH3 (EC_50_: 3.343 nM) was found to be the most effective in activating GnRHR1 compared to GnRH1 (EC_50_: 923 nM) and GnRH2 (EC_50_: 104.4 nM). In addition, GnRH2 (EC_50_: 0.61 nM) showed higher potency for GnRHR3 than GnRH1 (EC_50_: 2283 nM) and GnRH3 (EC_50_: 205.2 nM) (**Figure 2A**). Interestingly, GnRH1 (EC_50_: 267.4 nM), GnRH2 (EC_50_: 2.2 nM), and GnRH3 (EC_50_: 9.9 nM) all displayed high potency for GnRHR4, which suggested that GnRHR4 acted as a universal receptor for GnRHs in grass carp (**Figure 2A**). Furthermore, human GnRHR had been used to examine the potency for GnRHs. The result revealed that human GnRH1 could highly activate hGnRHR with EC EC_50_: 14.2 nM for NFAT-Luc and 10 nM for CRE-Luc, respectively. In addition, grass carp GnRH2 (EC_50_: 994.9 nM for NFAT-Luc and 54 nM for CRE-Luc) and GnRH3 (EC_50_: 54 nM for NFAT-Luc and 24.7 nM for CRE-Luc) also had a high activating potency for hGnRHR *via* the Ca^2+^ and PKA pathway (**Figure 2B**).

### Transcriptomic analysis of the pituitary actions of GnRH3 in grass carp

**Figure 3:**
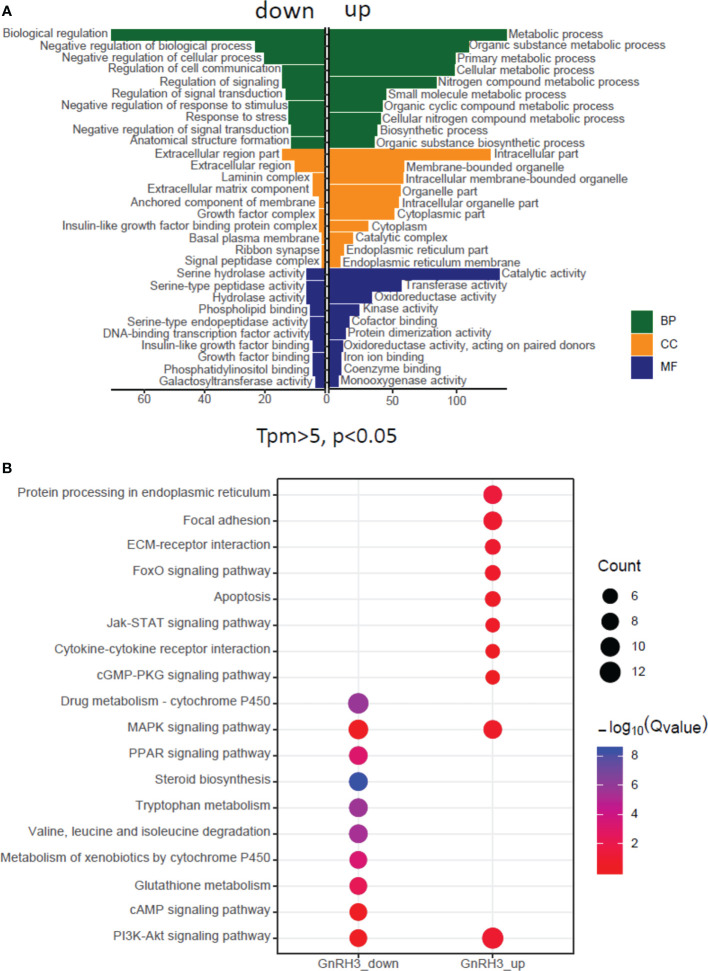
Gene Ontology (GO) analysis and Kyoto Encyclopedia of Genes and Genomes **(KEGG)** analysis based on transcriptome. **(A)** GO analysis revealed that the selected DEGs which satisfied the conditions of TPM >5, FDR <0.05, FC >1.5, or FC <0.7 were made up of three parts, namely, cellular component (CC), biological process (BP), and molecular function (MF). **(B)** One hundred sixty-two DEGs were classified into the top 10 enriched pathways using KEGG analysis which included upregulation and downregulation DEGs. Qvalue, Recalibration of P value. Count, the number of DEGs.

**Figure 4:**
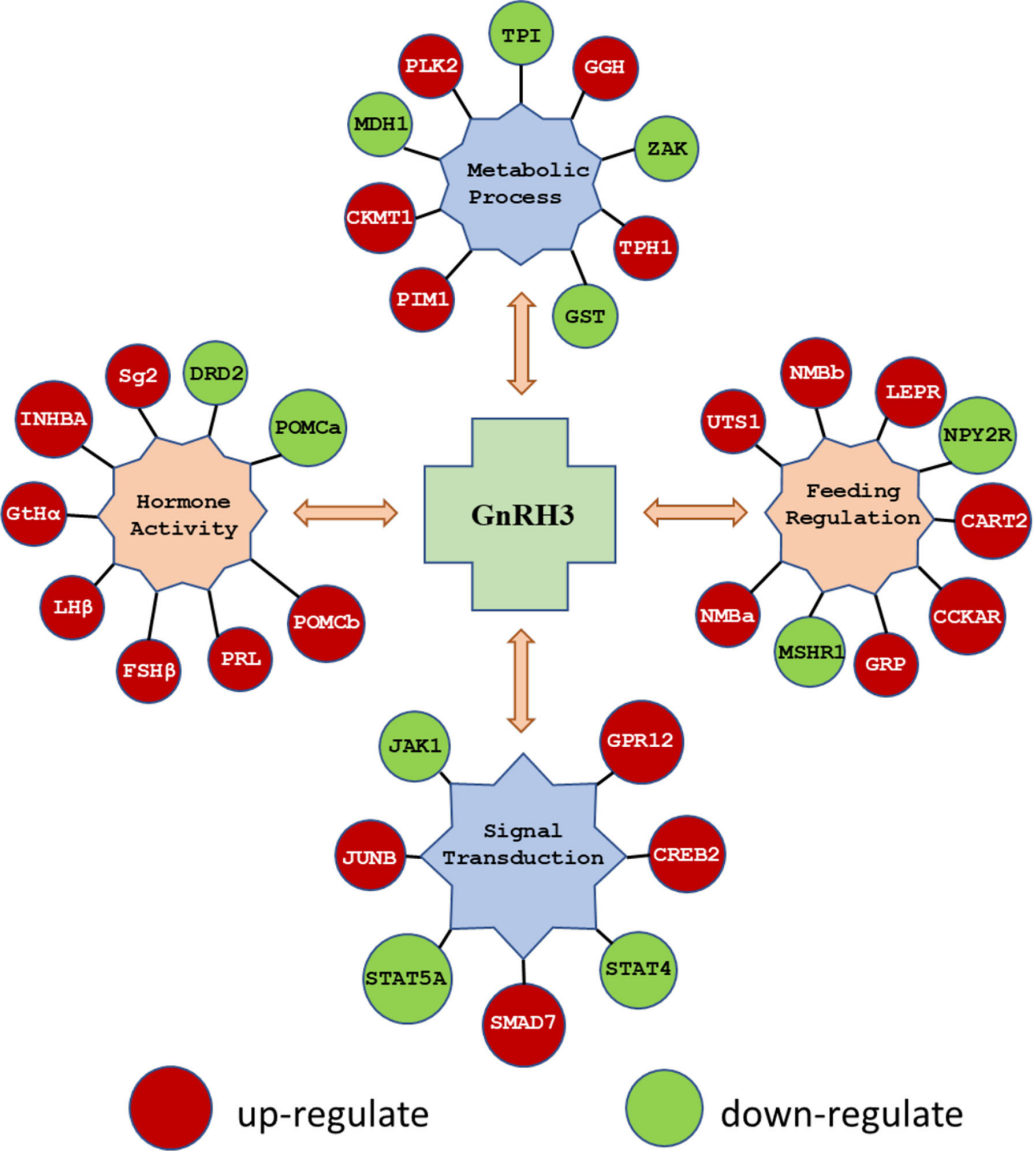
The key DEGs were selected to display the regulation of GnRH3 in signal transduction, feeding regulation, hormone activity, and metabolic process. The DEGs in red bubbles indicated the upregulated genes, and the DEGs in green bubbles indicated the downregulated genes by GnRH3 in grass carp pituitary cells.

In the present study, using grass carp pituitary cells as model, high-throughput RNA-seq was used to examine the pituitary actions of GnRH3 in teleost. According to the transcripts per kilobase of exon model per million mapped (TPM) reads method, a total of 820 different expression genes (DEGs) were screened under the condition of TPM >5, FDR <0.05. Subsequently, 245 upregulated genes (FC >1.5) and 575 downregulated genes (FC <0.7) were filtrated and used to perform GO analysis. The results showed that these DEGs were divided into three main ontologies, namely, cellular component, biological process, and molecular function (**Figure 3A**). The most abundant GO terms among the cellular component category were ‘intracellular part’, ‘membrane-bounded organelle’, ‘intracellular membrane-bounded organelle’, ‘organelle part’, and ‘intracellular organelle part’. Furthermore, the plentiful groups of biological processes were ‘metabolic process’, ‘organic substance metabolic process’, ‘primary metabolic process’, ‘cellular metabolic process’, and ‘biological regulation (**Figure 3A**). In addition, the GO enrichment analysis of molecular function was divided into two main contents, namely, 41 pivotal upregulated DEGs (**Table 1**) and 39 pivotal downregulated DEGs (**Table 2**). The Kyoto Encyclopedia of Genes and Genomes (KEGG) analysis showed that a total of 162 DEGs were enriched in the top 10 pathways. Among them, the upregulated DEGs were mostly enriched in ‘PI3K–Akt signaling pathway’ and ‘Focal adhesion’ and the downregulated DEGs were mainly enriched in ‘Drug metabolism-cytochrome P450’, ‘MAPK signaling pathway’, and ‘PPAR signaling pathway’ (**Figure 3B**). Finally, several key DEGs were selected to display the regulation of GnRH3 in signal transduction, feeding regulation, hormone activity, and metabolic process (**Figure 4**). In feeding regulation, several key genes such as proopiomelanocortin B (POMCb), cocaine and amphetamine-regulated transcript 2 (CART2), urotensin 1 (UTS1), neuromedin-B a (NMBa), neuromedin-B b (NMBb), and leptin receptor (LEPR) were selected and used as target genes on the detection of primary pituitary cells. In addition, several reproductive hormone genes, such as LHβ, FSHβ, glycoprotein hormones alpha (GtHα), inhibin-beta A (INHBA), secretogranin II, and dopamine D2 receptor (DRD2), were all regulated by GnRH3 and SgII (**Figure 4**).

**Table 1:** Upregulated genes by GnRH3 in grass carp pituitary cells.

Gene	FC	FDR	Description	Molecular Function
*tph1*	17.70	0	Tryptophan 5-hydroxylase 1	Amino acid binding
*bip*	1.78	5.39E-31	GRP78	ATP binding
*pim1*	3.25	0	threonine-protein kinase pim-1	ATP binding
*plk2*	5.78	0	Serine/threonine-protein kinase PLK2	ATP binding
*mcfd2*	1.65	3.57E-236	Multiple coagulation factor deficiency protein	Calcium ion binding
*nucb1*	1.56	1.72E-149	Nucleobindin-1 precursor	Calcium ion binding
*anx11*	3.17	0	Annexin 11a	Calcium ion binding
*nfasc*	10.71	5.61E-52	Neurofascin	Cell–cell adhesion mediator activity
*npr3*	11.53	0	Atrial natriuretic peptide receptor 3 isoform X1	Chloride ion binding
*e2.7.3.2*	3.88	0	Brain creatine kinase b	Creatine kinase activity
*ctsl*	2.55	0	Cathepsin La isoform X1	Cysteine-type peptidase activity
*scg2*	1.51	0	Secretogranin II precursor	Cytokine activity
*lepr*	2.60	6.73E-61	Leptin receptor long form	Cytokine binding
*grp*	1.52	1.46E-176	Gastrin-releasing peptide-like	Enzyme binding
*fth1*	1.90	0	Ferritin heavy chain	Ferric iron binding
*gpr61*	7.06	2.17E-78	Probable G-protein coupled receptor-like	G-protein-coupled receptor activity
*manf*	1.71	6.58E-124	Astrocyte-derived neurotrophic factor	Growth factor activity
*rergl*	6.29	2.35E-26	Estrogen-regulated inhibitor-like protein	GTP binding
*nmb*	3.29	4.39E-07	Neuromedin-B-like	Hormone activity
*crh*	1.74	6.72E-06	Urotensin 1	Hormone activity
*tsg6*	5.12	1.99E-06	Tumor necrosis factor-inducible gene 6 protein	Hyaluronic acid binding
*il10ra*	27.19	0.009871	Interleukin-10 receptor 1	Interleukin-10 binding
*tt39b*	6.76	6.74E-06	Tetratricopeptide repeat protein 39B-like	Lipid metabolic process
*agrb1*	8.87	0	Brain-specific angiogenesis inhibitor 1-like	Lipopolysaccharide binding
*zbtb8*	1.69	1.08E-73	Zinc finger domain-containing protein 8A	Metal ion binding
*ctro*	5.24	3.29E-164	Citron Rho-interacting kinase	Metal ion binding
*sdf2l*	1.55	1.30E-57	Stromal cell-derived factor 2-like 1	Misfolded protein binding
*cart2*	19.16	1.73E-22	Amphetamine-regulated transcript II precursor	Neuropeptide hormone activity
*ggh*	1.51	1.06E-186	Gamma-glutamyl hydrolase	Omega peptidase activity
*pdyn*	5.14	7.38E-17	Proenkephalin-B-like isoform X1	Opioid receptor binding
*spcs3*	1.77	2.74E-239	Signal peptidase complex subunit 3	Peptidase activity
*fkbp11*	1.94	0	Peptidyl-prolyl cis-trans isomerase	cis-trans isomerase activity
*r4rl2*	93.68	6.85E-299	Reticulon 4 receptor-like 2b precursor	Protein binding
*bha15*	1.66	0	Class A basic helix-loop-helix protein 15	Protein dimerization activity
*junb*	1.89	2.36E-96	Transcription factor jun-B	Sequence-specific DNA binding
*nec1*	1.54	0	Neuroendocrine convertase 1	Serine-type endopeptidase activity
*hpt*	5.16	4.16E-131	Haptoglobin-like	Serine-type endopeptidase activity
*sec11*	1.55	1.55E-131	Signal peptidase complex catalytic subunit	Serine-type peptidase activity
*nab1*	5.88	3.65E-131	NGFI-A-binding protein 1	Transcription factor binding
*rdl*	1.57	3.51E-111	Thiosulfate sulfurtransferase	Transferase activity
*sqstm1*	1.69	0	Sequestosome-1 isoform X1	Zinc ion binding
*fsh*	1.35	6.77E-14	Follicle stimulating hormone	Hormone activity
*lhβ*	1.32	0	Luteinizing hormone	Hormone activity
*gthα*	1.28	0	Glycoprotein hormones alpha chain	Hormone activity
*inhba*	1.90	1.08E-11	Inhibin-beta A	Growth factor activity
*SgII*	1.31	2.73E-241	Secretogranin II	Calcium ion binding
*prl*	1.22	0	Prolactin	Hormone activity
*cckar*	1.41	0.40	Cholecystokinin receptor-like isoform	G protein-coupled receptor activity
*creb2*	1.56	1.69E-07	Activating transcription factor 4b	cAMP response element binding protein binding
*smad7*	1.78	7.44E-31	Mothers against decapentaplegic homolog 7	Beta-catenin binding

FC, fold change; FDR, false discovery rate.

**Table 2:** Downregulated genes by GnRH3 in grass carp pituitary cells.

Gene	FC	FDR	Description	Molecular function
*arpc4*	0.66	1.86E-42	Actin-related protein 2/3 subunit 4-like	Actin binding
*nlrp3*	0.26	0.000497	PYD domains-containing protein 3-like	ATP binding
*chd4*	0.64	3.30E-172	Chromodomain-DNA-binding protein 4	Binding
*calm*	0.68	2.37E-221	Calmodulin	Calcium ion binding
*marcksl1b*	0.67	2.63E-101	Myristoylated alanine-rich C substrate 2	Calmodulin binding
*hint1*	0.66	7.80E-16	Histidine triad nucleotide-binding protein 1	Catalytic activity
*dgkz*	0.41	0.003213	Diacylglycerol kinase zeta-like isoform X1	Diacylglycerol kinase activity
*ppp1r10*	0.42	2.43E-39	Threonine-protein phosphatase 1 regulatory	DNA binding
*hmgb1*	0.69	7.95E-46	High-mobility group box 1	DNA binding
*rfx1*	0.28	0	MHC class II regulatory factor RFX1	DNA-binding factor activity
*fabp7*	0.50	5.12E-107	Fatty acid binding protein 7	Fatty acid binding
*gst*	0.67	8.59E-06	Glutathione S-transferase	Glutathione transferase activity
*gmpr1*	0.61	2.64E-126	GMP reductase 1	GMP reductase activity
*rhoa*	0.32	0.042946	Transforming protein rhoa	GTP binding
*mx1*	0.29	0.004203	Antiviral effector Mx3	GTPase activity
*h4*	0.68	0.002615	Histone H4	Histone demethylase activity
*gphb5*	0.69	2.13E-64	Glycoprotein hormone beta-5	Hormone activity
*atp5me*	0.69	5.40E-07	ATP synthase subunit e	Hydrolase activity
*erg7*	0.43	1.00E-62	Lanosterol synthase	Intramolecular activity
*mdh1*	0.68	4.31E-77	Cytosolic malate dehydrogenase	L-malate dehydrogenase activity
*m3k20*	0.31	0.00526	Mitogen-activated protein kinase kinase 20	MAP kinase kinase activity
*s100b*	0.31	3.01E-240	Protein S100-B	Metal ion binding
*cdc42bp*	0.37	0.004184	Serine/threonine-protein kinase MRCK beta	Metal ion binding
*zfp36l*	0.66	1.92E-125	Zinc finger protein 36, C3H type-like 1b	Metal ion binding
*ppp1r17*	0.68	2.87E-10	Protein phosphatase 1 regulatory 17-like	Phosphatase inhibitor activity
*pa2g3*	0.29	6.47E-212	Group 3 secretory phospholipase A2-like	Phospholipase A2 activity
*snf8*	0.44	1.18E-06	Vacuolar-sorting protein SNF8-like	Protein binding
*h2b3*	0.53	4.58E-23	Histone H2B 3	Protein heterodimerization activity
*h2a*	0.63	1.56E-20	Histone H2A	Protein heterodimerization activity
*mvp*	0.64	2.14E-255	Major vault protein	Ribonucleoprotein
*estd*	0.37	4.80E-36	S-formylglutathione hydrolase	S-Formylglutathione activity
*sstr3*	0.42	3.09E-126	Somatostatin receptor type 3	Somatostatin receptor activity
*tubb*	0.40	3.79E-49	Tubulin beta-4B chain	Structural constituent of cytoskeleton
*suhb*	0.44	2.86E-22	3-Beta-hydroxysteroid sulfotransferase	Sulfotransferase activity
*sod1*	0.69	1.09E-31	Copper/zinc superoxide dismutase	Superoxide dismutase activity
*nltp*	0.41	2.86E-12	Non-specific lipid-transfer protein	Transferase activity
*fabp3*	0.63	1.56E-177	Fatty-acid binding protein 3b	Transporter activity
*tpisb*	0.68	8.61E-53	Triosephosphate isomerase B	Triose-phosphate isomerase activity
*vdac3*	0.69	5.87E-44	Voltage-dependent anion-selective protein 3	Voltage-gated anion channel activity
*drd2*	0.35	2.63E-37	Dopamine D2 receptor	G-protein coupled receptor activity
*pomca*	0.90	0	Pro-opiomelanocortin I	Neuropeptide signaling pathway
*zak*	0.31	0.00526009	Mitogen-activated protein kinase kinase 20	Catalytic Activity
*npy2r*	0.44	0.07342027	Europeptide Y receptor type 2-like	Peptide YY receptor activity
*mshr1*	0.43	0	Melanocyte-stimulating hormone receptor	Hormone receptor activity
*stat5a*	0.89	0.04174356	Stat5.2 protein	DNA-binding factor activity
*stat4*	0.86	9.86E-21	Stat 4-like	Binding to a chemokine receptor

FC, fold change; FDR, false discovery rate.

### Regulation of reproductive hormones genes by different GnRH variants in grass carp pituitary cells

**Figure 5:**
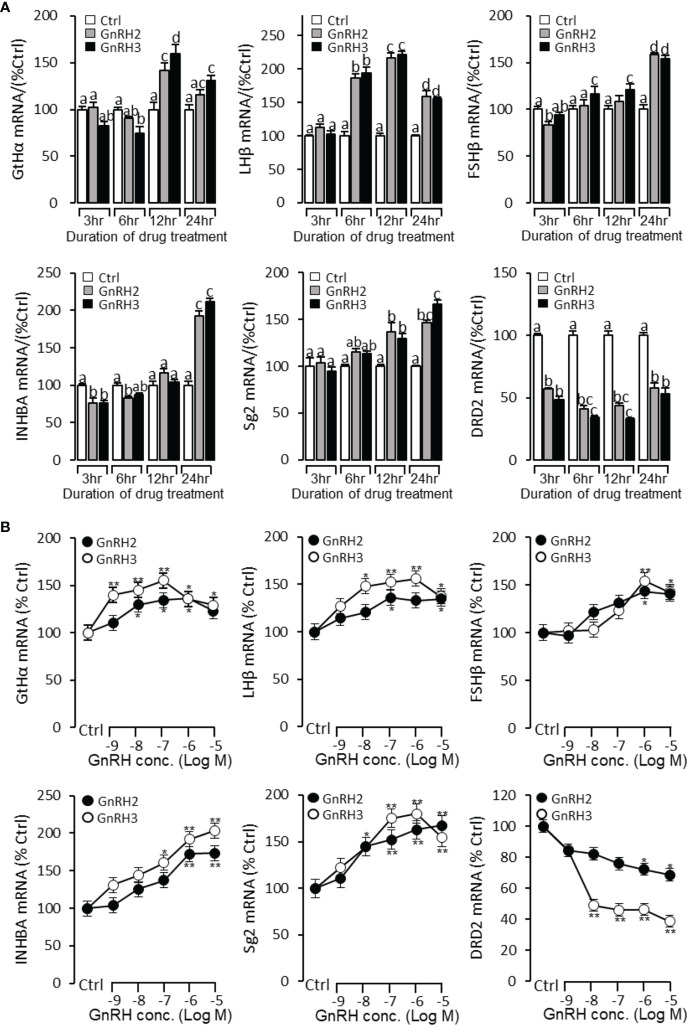
Regulation of reproductive hormone gene mRNA expression by GnRH2 and GnRH3 in grass carp pituitary cells. **(A)** Time-course experiment of grass carp GnRH2 (1 μM) and GnRH3 (1 μM) on GtHα, LHβ, FSHβ, INHBa, SgII, and DRD2 mRNA expression in grass carp pituitary cells. **(B)** Dose dependence of a 24-h treatment with increasing levels of GnRH2 and GnRH3 (0.1–1,000 nM) on GtHα, LHβ, FSHβ, INHBa, SgII, and DRD2 mRNA expression in grass carp pituitary cells. After drug treatment, the pituitary cells were extracted to total RNA, reversed transcribed, and used for RT-PCR to detect the target genes’ mRNA expression. Data presented are expressed as mean ± SEM. *p* < 0.05 (“*”) or *p* < 0.01 (“**”) was used to present significant differences among each group. The different letters represent a significant difference at *p* < 0.05 between groups (ANOVA followed by a Dunnett test).

To further verify the reproductive function of GnRHs, time- and dose-dependent tests were performed to detect GnRH2- or GnRH3-regulated GtHα, LHβ, FSHβ, INHBa, SgII, and DRD2 mRNA expression in grass carp pituitary cells. The time-course test showed that both GnRH2 (1 μM) and GnRH3 (1 μM) could significantly induce pituitary GtHα, LHβ, FSHβ, INHBa, and SgII mRNA expression and inhibit pituitary DRD2 mRNA expression in a time-dependent manner (**Figure 5A**). In the dose-dependent test, a continuous gradient dilution of GnRH2 (0.1–1,000 nM) or GnRH3 (0.1–1,000 nM) was incubated with grass carp pituitary cells for 24 h. The results revealed that both GnRH2 and GnRH3 could significantly induce GtHα, LHβ, FSHβ, INHBa, and SgII mRNA expression in a dose-dependent manner (**Figure 5B**). In addition, all treated doses of GnRH2 and GnRH3 could significantly inhibit DRD2 mRNA expression (**Figure 5B**).

### Signal transduction for GnRH-regulated reproductive hormone gene mRNA expression in grass carp pituitary cells

**Figure 6:**
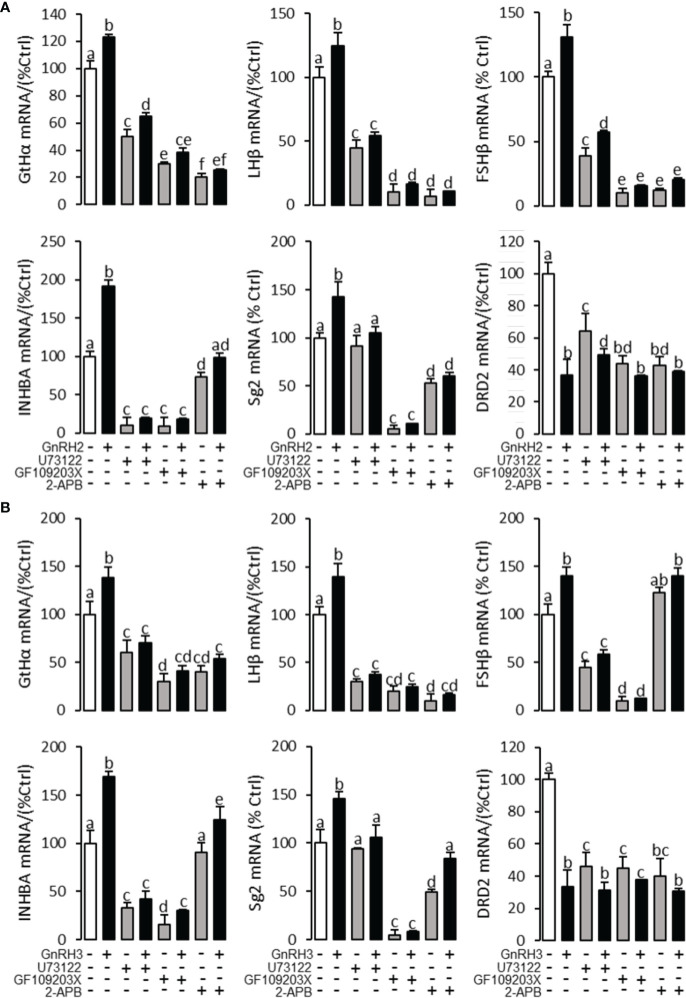
Signal transduction for GnRH2- and GnRH3-regulated GtHα, LHβ, FSHβ, INHBa, SgII, and DRD2 mRNA expression in grass carp pituitary cells. In this experiment, GnRH2 **(A)** or GnRH3 **(B)** combined with**/**without the PLC inhibitor U73122 (10 μM), PKC inhibitor GF109203X (20 μM), or IP3 receptor blocker 2-APB (100 μM) was used to incubate the grass carp pituitary cells for 24 (h) After drug treatment, the total RNA was extracted from the cells for real-time PCR of the respective genes. Data presented are expressed as mean ± SEM, and the different letters represent a significant difference at *p* < 0.05 between groups (ANOVA followed by a Dunnett test).

**Figure 7:**
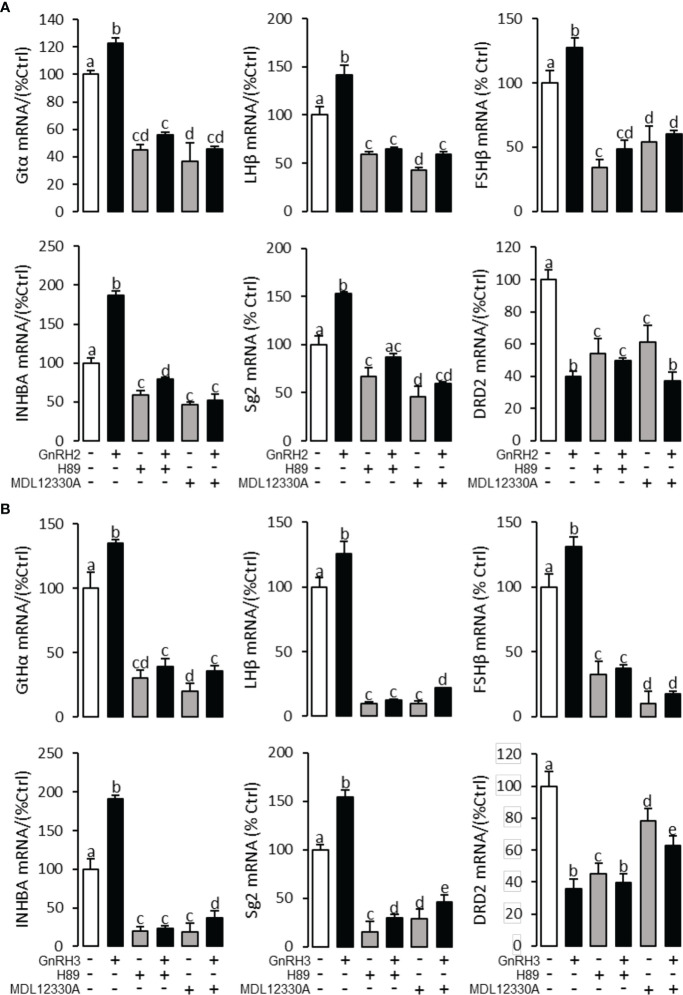
Signal transduction for GnRH2- and GnRH3-regulated GtHα, LHβ, FSHβ, INHBa, SgII, and DRD2 mRNA expression in grass carp pituitary cells. In this experiment, GnRH2 **(A)** or GnRH3 **(B)** combined with**/**without the AC inhibitor MDL12330A (20 μM) or PKA inhibitor H89 (20 μM) was used to incubate the grass carp pituitary cells for 24 (h) After drug treatment, the total RNA was extracted from the cells for real-time PCR of the respective genes. Data presented are expressed as mean ± SEM, and the different letters represent a significant difference at *p* < 0.05 between groups (ANOVA followed by a Dunnett test).

**Figure 8:**
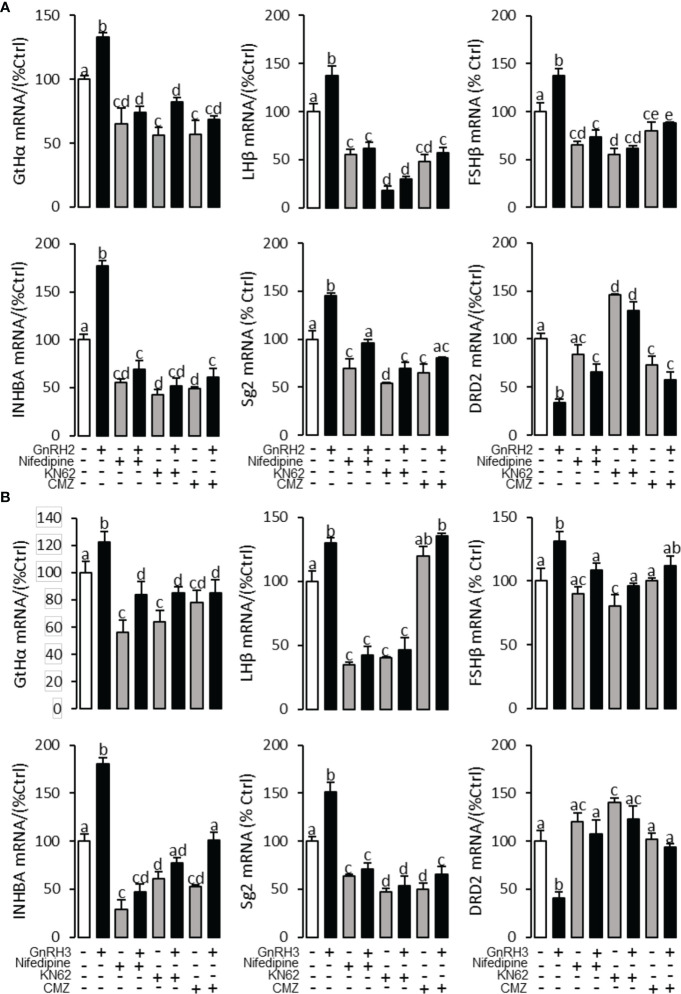
Signal transduction for GnRRH2- and GnRH3-induced GtHα, LHβ, FSHβ, INHBa, SgII, and DRD2 mRNA expression in grass carp pituitary cells. In this experiment, GnRH2 **(A)** or GnRH3 **(B)** combined with**/**without the VSCC inhibitor nifedipine (10 μM), CaM antagonist calmidazolium (1 μM), or CaMK-II blocker KN62 (5 μM) was used to incubate the grass carp pituitary cells for 24 (h) After drug treatment, the total RNA was extracted from the cells for real-time PCR of the respective genes. Data presented are expressed as mean ± SEM, and the different letters represent a significant difference at *p* < 0.05 between groups (ANOVA followed by a Dunnett test).

To explore the signal transduction for GnRH-regulated reproductive hormone gene mRNA expression, the inhibitors of AC/PKA, PLC/PKC, and IP_3_/Ca^2+^ signal pathways were cotreated with GnRH2 or GnRH3 in grass carp pituitary cells. The results revealed that PLC inhibitor U73122 (10 μM), PKC inhibitor GF109203X (20 μM), and IP3 receptor blocker 2-APB (100 μM) could block GnRH2- (**Figure 6A**) or GnRH3- (**Figure 6B**) induced GtHα, LHβ, FSHβ, INHBa, and SgII mRNA expression in grass carp pituitary cells, respectively. In addition, the stimulatory effects of GnRH2 (**Figure 7A**) or GnRH3 (**Figure 7B**) on GtHα, LHβ, FSHβ, INHBa, and SgII mRNA expression could also be blocked by AC inhibitor MDL12330A (20 μM) or PKA inhibitor H89 (20 μM) in grass carp pituitary cells. Furthermore, the VSCC inhibitor nifedipine (10 μM), CaM antagonist calmidazolium (1 μM), or CaMk-II blocker KN62 (5 μM) could also block GnRH2- (**Figure 8A**) or GnRH3-(**Figure 8B**) induced GtHα, LHβ, FSHβ, INHBa, and SgII mRNA expression. Interestingly, GnRH2- or GnRH3-reduced pituitary DRD2 mRNA expression could not be blocked by PLC/PKC inhibitors (**Figure 6**) or AC/PKA inhibitors (**Figure 7**). However, the inhibitors for the Ca^2+^/CaM/CaM-II cascade could significantly block GnRH2- (**Figure 8A**) or GnRH3- (**Figure 8B**) reduced DRD2 mRNA expression in grass carp pituitary cells.

### Regulation of six anorectic genes’ (POMCb, CART2, UTS1, NMBa, NMBb, and LEPR) mRNA expression by GnRH2 and GnRH3 in grass carp pituitary cells

**Figure 9:**
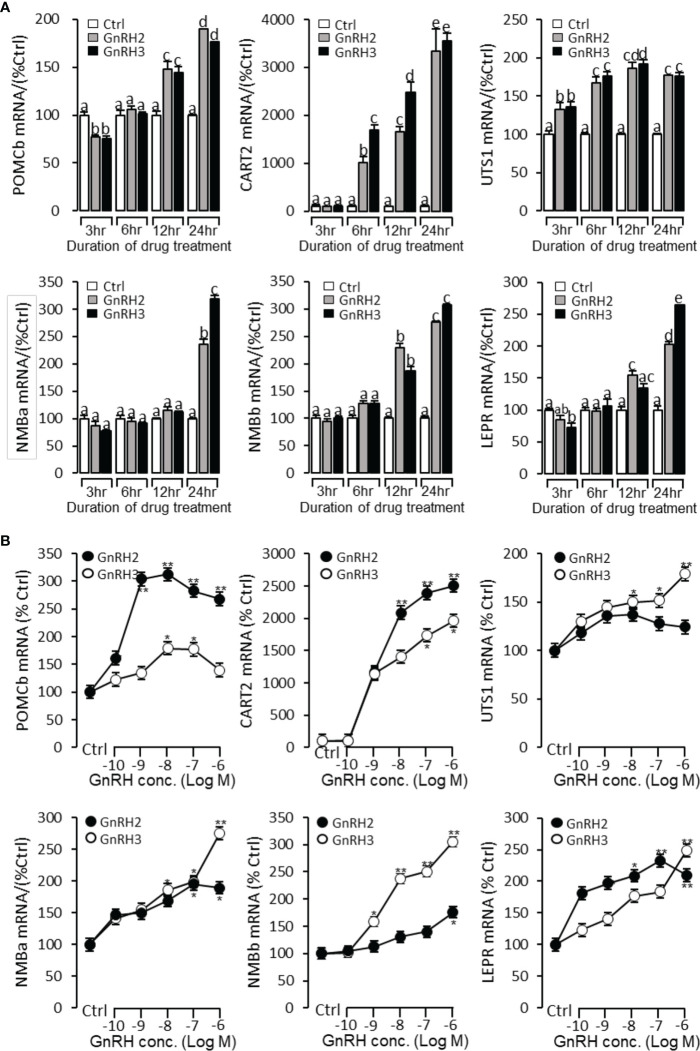
Regulation of feeding hormone gene mRNA expression by GnRH2 and GnRH3 in grass carp pituitary cells. **(A)** Time-course experiment of grass carp GnRH2 (1 μM) and GnRH3 (1 μM) on POMCb, CART2, UTS1, NMBa, NMBb, and LEPR mRNA expression in grass carp pituitary cells. **(B)** Dose dependence of a 24-h treatment with increasing levels of GnRH2 and GnRH3 (0.1–1,000 nM) on POMCb, CART2, UTS1, NMBa, NMBb, and LEPR mRNA expression in grass carp pituitary cells. After drug treatment, the pituitary cells were extracted to total RNA, reverse transcribed, and used for RT-PCR to detect the target genes’ mRNA expression. Data presented are expressed as mean ± SEM. *p* < 0.05 (“*”) or *p* < 0.01 (“**”) was used to present significant differences among each group. The different letters represent a significant difference at *p* < 0.05 between groups (ANOVA followed by a Dunnett test).

To further verify the functional role of GnRH2 and GnRH3 on feeding regulation, time- and dose-dependent tests were performed to detect the effect of GnRH2 and GnRH3 on anorectic gene (POMCb, CART2, UTS1, NMBa, NMBb, and LEPR) mRNA expression. In the time-course test, the grass carp pituitary cells were incubated by GnRH2 or GnRH3 (1 μM) for 3, 6, 12, and 24 h, respectively. The results showed that both GnRH2 and GnRH3 could significantly induce POMCb, CART2, UTS1, NMBa, NMBb, and LEPR mRNA expression in a time-dependent manner (**Figure 9A**). In the dose-dependent test, grass carp pituitary cells were incubated by the continuous gradient dilution of GnRH2 or GnRH3 (0.1–1,000 nM) for 24 h. The results revealed that both GnRH2 and GnRH3 could significantly induce POMCb, CART2, UTS1, NMBa, NMBb, and LEPR mRNA expression in a dose-dependent manner (**Figure 9B**).

### Signal transduction for GnRH-induced POMCb, CART2, UTS1, NMBa, NMBb, and LEPR mRNA expression in grass carp pituitary cells

**Figure 10:**
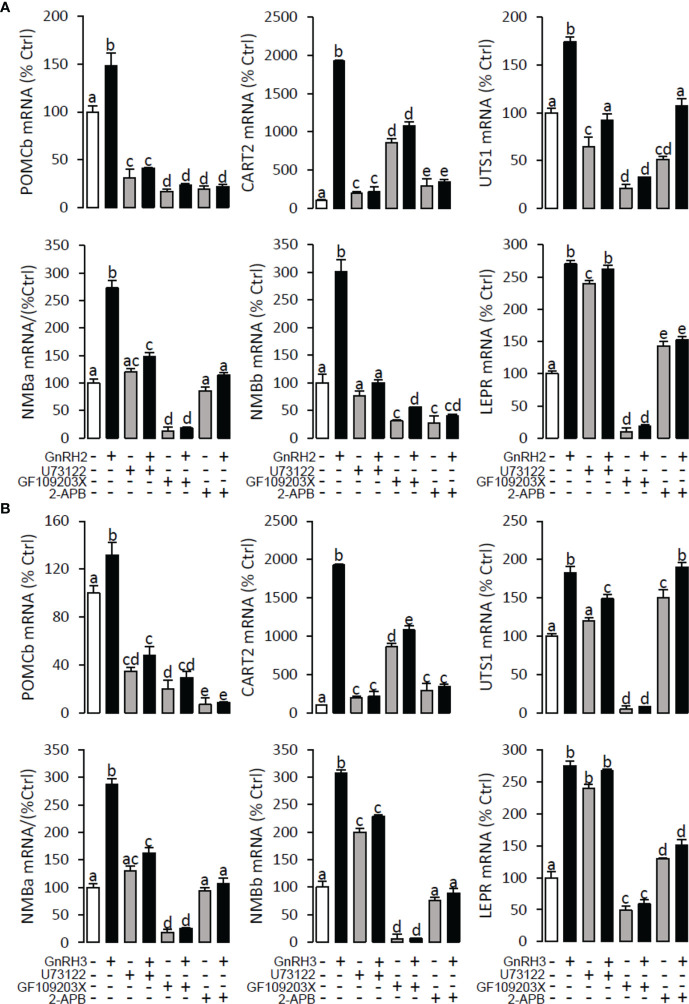
Signal transduction for GnRH2- and GnRH3-induced POMCb, CART2, UTS1, NMBa, NMBb, and LEPR mRNA expression in grass carp pituitary cells. In this experiment, GnRH2 **(A)** or GnRH3 **(B)** combined with**/**without the PLC inhibitor U73122 (10 μM), PKC inhibitor GF109203X (20 μM), or IP3 receptor blocker 2-APB (100 μM) was used to incubate the grass carp pituitary cells for 24 (h) After drug treatment, the total RNA was extracted from the cells for real-time PCR of the respective genes. Data presented are expressed as mean ± SEM, and the different letters represent a significant difference at *p* < 0.05 between groups (ANOVA followed by a Dunnett test).

**Figure 11:**
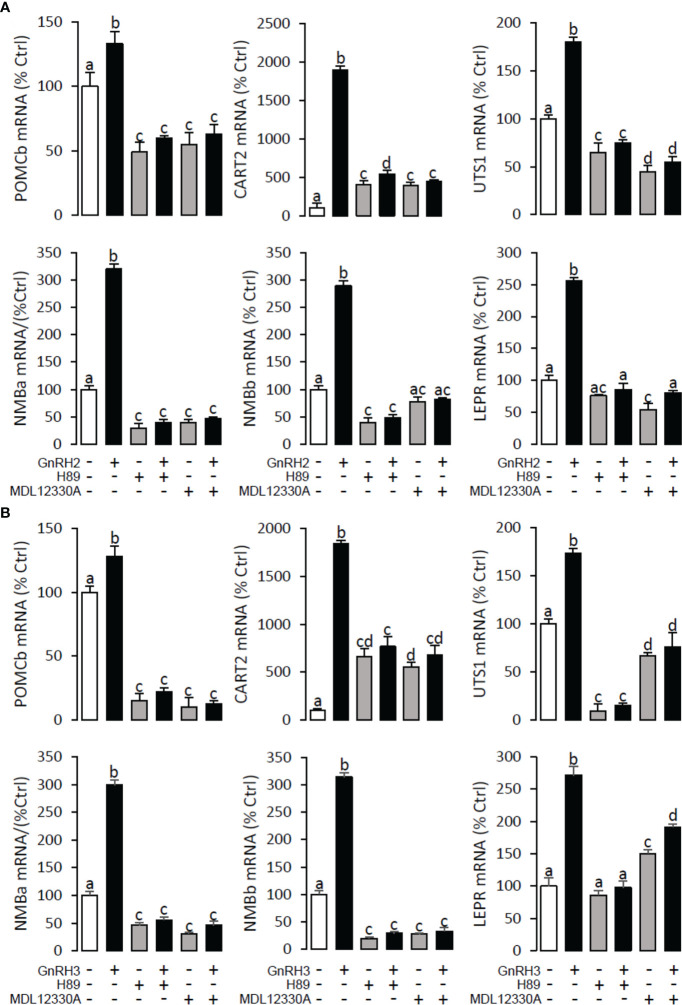
Signal transduction for GnRH2- and GnRH3-induced POMCb, CART2, UTS1, NMBa, NMBb, and LEPR mRNA expression in grass carp pituitary cells. In this experiment, GnRH2 **(A)** or GnRH3 **(B)** combined with**/**without the AC inhibitor MDL12330A (20 μM) or PKA inhibitor H89 (20 μM) was used to incubate the grass carp pituitary cells for 24 (h) After drug treatment, the total RNA was extracted from the cells for real-time PCR of the respective genes. Data presented are expressed as mean ± SEM, and the different letters represent a significant difference at *p* < 0.05 between groups (ANOVA followed by a Dunnett test).

**Figure 12:**
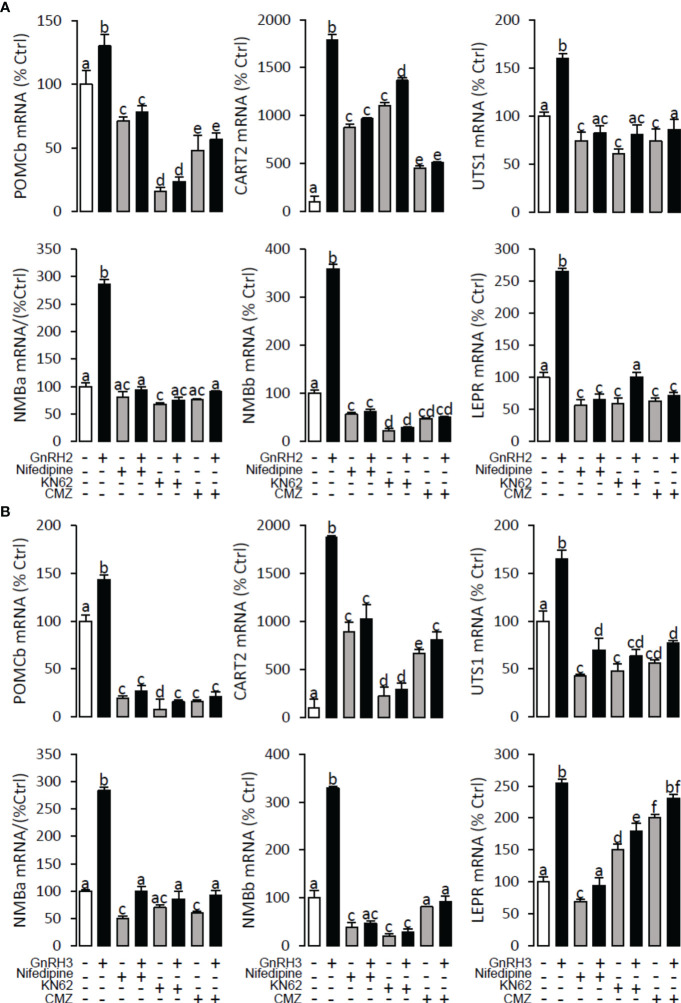
Signal transduction for GnRH2- and GnRH3-induced POMCb, CART2, UTS1, NMBa, NMBb, and LEPR mRNA expression in grass carp pituitary cells. In this experiment, GnRH2 **(A)** or GnRH3 **(B)** combined with**/**without the VSCC inhibitor nifedipine (10 μM), CaM antagonist calmidazolium (1 μM), or CaMK-II blocker KN62 (5 μM) were used to incubate the grass carp pituitary cells for 24 (h) After drug treatment, the total RNA was extracted from the cells for real-time PCR of the respective genes. Data presented are expressed as mean ± SEM, and the differences between groups were significant at *p* < 0.05 by labelling diverse letters (ANOVA followed by a Dunnett test).

To explore the signal transduction for GnRH-induced target gene mRNA expression, the method mentioned above was performed. The results revealed that PLC inhibitor U73122 (10 μM), PKC inhibitor GF109203X (20 μM), IP3 receptor blocker 2-APB (100 μM) (**Figure 10**), AC inhibitor MDL12330A (20 μM), PKA inhibitor H89 (20 μM) (**Figure 11**), VSCC inhibitor nifedipine (10 μM), CaM antagonist calmidazolium (1 μM), and CaMk-II blocker KN62 (5 μM) (**Figure 12**) could block both GnRH2- or GnRH3-induced target gene mRNA expression in grass carp pituitary cells, respectively.

### Postprandial changes in brain GnRH2 and GnRH3 expression after food intake in grass carp

**Figure 13:**
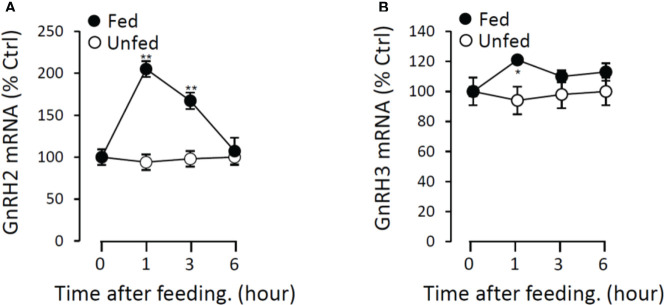
Postprandial changes of GnRH2 **(A)** and GnRH3 **(B)** mRNA expression in grass carp. Grass carp were temporarily raised in a well-aerated 250-l storage pond and fed one meal per day for at least 7 days on fixed times (9:00 a.m.). The brains were harvested at 0, 1, 3, and 6 h after food administration. Then, the total RNA were extracted to detect the GnRH mRNA level expression by the RT-PCR system. Data presented are expressed as mean ± SEM, and the differences between groups were significant at *p* < 0.05 by labeling symbol “*” (“**” means *p* < 0.01).

To further verify the potential functional role of GnRHs on feeding regulation, the expressions of GnRH2 and GnRH3 mRNA in grass carp brain were monitored after the meal. In the control group without feeding (unfed group), the transcript levels of GnRH2 and GnRH3 in the brain did not change from 1 to 6 h. In contrast, food intake could significantly induce brain GnRH2 and GnRH3 mRNA expression with a peak at 1 h (**Figure 13**).

## Discussion

In mammal, only one or two GnRHR variants have been identified (23). However, five GnRHRs have been detected in teleosts, such as European seabass (24) and pufferfish (25). Similar to zebrafish (26), four GnRH receptors (namely, GnRHR1, GnRHR2, GnRHR3, GnRHR4) have been cloned in grass carp. In the present study, by using transfection and luciferase assay, we found that individual subtypes of GnRHR exhibited differential selectivity for various members of GnRHs, with GnRHR3 preferring for GnRH2, and GnRHR1 preferring for GnRH3, respectively. Interestingly, grass carp GnRHR4 could display a rank order of GnRH1≈GnRH2≈GnRH3 for receptor activation, which suggested that GnRHR4 should be a multiligand receptor with promiscuity for three GnRHs. In grass carp, our previous study has found that NK2R was a multiligand receptor for various tachykinin peptides (15). In mammals, multiligand receptors with promiscuity for structurally related ligands (or even unrelated ligands) have been reported, for example, class A (27) and class B type I scavenger receptors (28), receptor for advanced glycation end product (29), and related protein for low-density lipoprotein receptor (30). The deviation from the “one ligand/one receptor” model for receptor activation is thought to have occurred during early evolution and allows for effective integration of extracellular signals mediated by ligands of the same family or even dissimilar ligands with related functions (31). In addition, the ligand–receptor selectivity experiment also showed that grass carp GnRHRs could be activated by human GnRH1 with comparable efficacy and potency. This is the reason why hGnRH1 could be used in artificial reproduction of grass carp. In addition, we also found that human GnRHR could also be activated by grass carp GnRH2 and GnRH3, suggesting that the GnRH/GnRHR system was very conserved from teleost to mammal.

In the present study, although the transcript level of GnRH2 was not detected in immature grass carp pituitary by RT-PCR, GnRH2 could also significantly induce LHβ and FSHβ mRNA expression in grass carp pituitary cells. Similarly, recent studies in zebrafish, a two-GnRH model species exhibiting two GnRH variants into the brain (GnRH2 and GnRH3), with a dominant pituitary presence of the hypophysiotropic GnRH3 (32), showed that under fasting conditions, GnRH3 disappeared from the pituitary, while the levels of GnRH2 increased (9, 33). In the two-GnRH goldfish model, GnRH2 elicited a more robust LH secretion compared to GnRH3 in sexually mature, pre-spawning fish, while in sexually regressed animals, GnRH3 had potent LH-releasing activity and GnRH2 had no effect (34, 35). These results indicated that GnRH2 may serve as a “backup” system to ensure the integrity of reproduction under suboptimal or other specific physiological conditions. In addition, we found that GnRH2 and GnRH3 could not only induce pituitary GtHα, LHβ, and FSHβ mRNA expression but also stimulate SgII mRNA expression in grass carp pituitary cells. A recent study has reported that mutation of SgII in zebrafish could lead to disrupted sexual behaviors, reduced ovulation and egg laying, and suboptimal fertility and embryo survival (36). These results suggested that GnRH could also regulate the reproduction through inducing SgII expression in teleost. Interestingly, we found that GnRHs could significantly reduce dopamine D2 receptor expression in grass carp pituitary cells. As we know, SgII dopamine inhibited LH synthesis *via* activation of DRD2 in teleost (37), suggesting that GnRH2 and GnRH3 could block dopamine-reduced LH expression by inhibiting pituitary DRD2 mRNA expression. After using the inhibitors of AC/PKA, PLC/PKC, and IP_3_/Ca^2+^ signal pathways to cotreat with GnRH2 or GnRH3 in grass carp pituitary cells, we found that these inhibitors could block GnRH2- or GnRH3-induced GtHα, LHβ, FSHβ, INHBa, and SgII mRNA expression.

For feeding regulation, it had been reported that intracerebroventricular (ICV) injections of GnRH2 could significantly decrease food intake in zebrafish (38) and goldfish (39). Besides, knockout of GnRH2 could observably increase the food intake in zebrafish (9). In the present study, postprandial GnRH*2* and GnRH3 mRNA expression increased in a short time after food intake, indicating that GnRHs could act as a transient anorexigenic peptide in grass carp. Subsequently, we found that POMCb (40), CART2 (41), UTS1 (42), NMBa, NMBb (43), and LEPR (44), which had been reported as anorexigenic peptides, could be significantly stimulated by GnRH2 and GnRH3 in grass carp pituitary cells. These results suggested that GnRHs should be the satiety factor and involved in the regulation of pituitary anorectic peptides in teleost.

**Figure 14:**
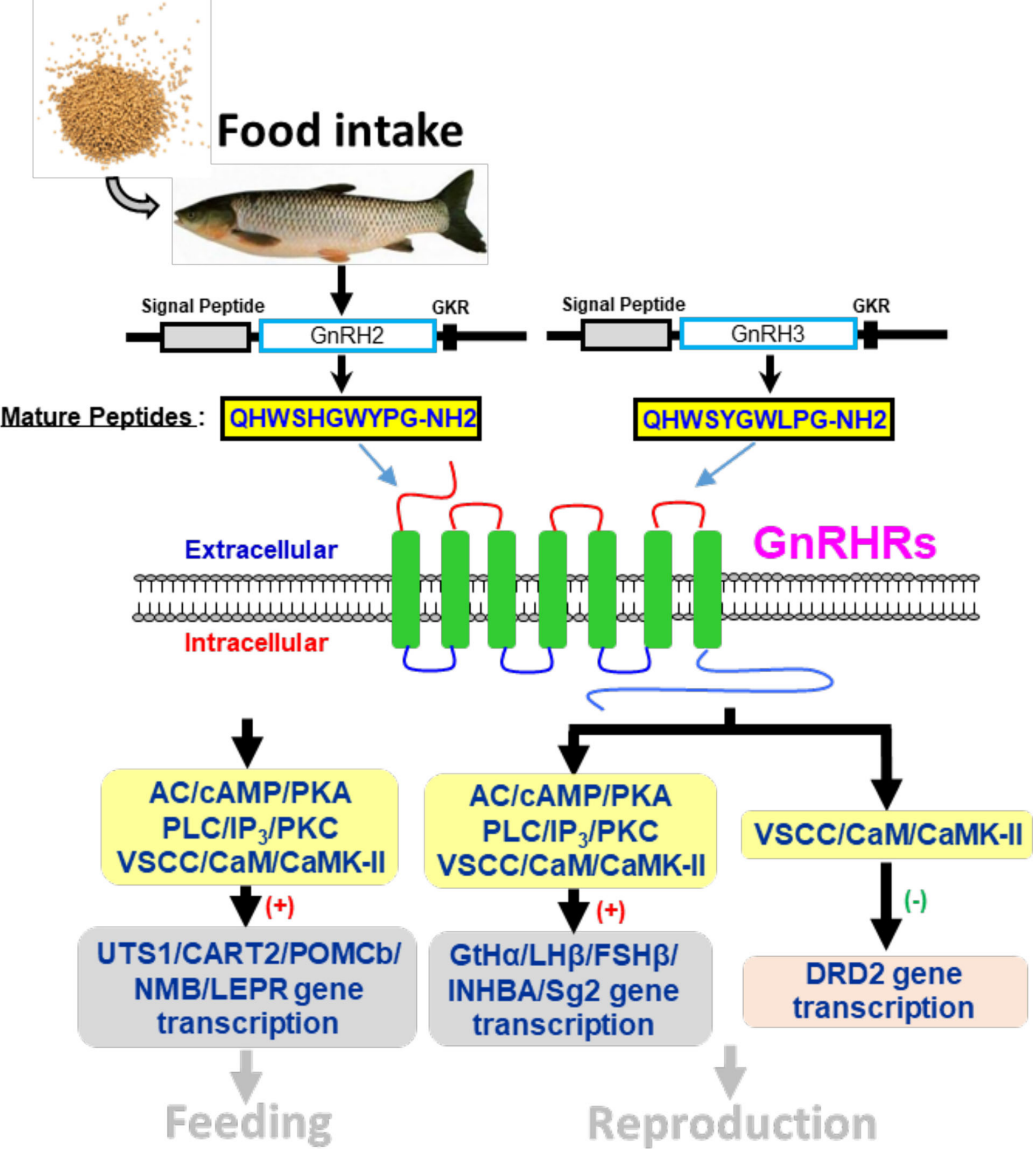
Working model of GnRH-mediated feeding and reproduction in grass carp. Two GnRH ligands and four GnRHRs were cloned from grass carp brain, and ligand–receptor selectivity showed that GnRHR3 was a specific receptor for GnRH2 when GnRH3 was preferentially chosen combined with GnRHR1, and GnRHR2 and GnRHR4 might be universal receptors to both GnRHs. Besides, GnRH2 and GnRH3 could both significantly induce pituitary reproductive hormone gene (GtHα, LHβ, FSHβ, INHBa, SgII) and feeding hormone gene (POMCb, CART2, UTS1, NMBa, NMBb, and LEPR) mRNA expression mediated by AC/PKA, PLC/IP3/PKC and Ca^2+^/CaM/CaMK-II pathways *in vitro*. Besides, GnRH2 and GnRH3 could modulate the inhibition of LH by dopamine according to a restrained DRD2 expression *via* the Ca^2+^/CaM/CaMK-II pathway. Finally, food intake could significantly induce brain GnRH2 and GnRH3 mRNA expression.

In summary, the two GnRH ligands (GnRH2 and GnRH3) and four GnRHRs (namely, GnRHR1, GnRHR2, GnRHR3, GnRHR4) were cloned from grass carp brain and pituitary. Then, ligand–receptor selectivity showed that individual subtypes of GnRHR exhibited differential selectivity for various members of GnRHs, with GnRHR3 preferring for GnRH2 and GnRHR1 preferring for GnRH3, respectively. Interestingly, GnRHR4 should be a multiligand receptor for GnRH2 and GnRH3. Using grass carp pituitary cells as model, we found that GnRH2 and GnRH3 could not only directly induce LHβ and FFSHβ mRNA expression but also stimulate other reproductive genes’ (INHBa and SgII) mRNA expression mediated by AC/PKA, PLC/IP3/PKC, and Ca^2+^/CaM/CaMK-II pathways. In addition, GnRH2 and GnRH3 could inhibit DRD2 mRNA expression to block dopamine-reduced LH secretion and synthesis. Finally, food intake could significantly induce brain GnRH2 and GnRH3 mRNA expression, and both GnRH2 and GnRH3 could significantly induce pituitary anorexigenic peptides’ (POMCb, CART2, UTS1, NMBa, and NMB*b*) mRNA expression *via* AC/PKA, PLC/IP3/PKC, and Ca^2+^/CaM/CaMK-II pathways (**Figure 14**). These results indicated that GnRHs could be involved in the regulation of reproduction and feeding.

## Data availability statement

The transcriptomic data presented in the study were deposited to NCBI repository https://www.ncbi.nlm.nih.gov/sra, accession number SRR21736247. In addition, the sequences of grass carp GnRHs and GnRHRs were also submitted to Genbank https://www.ncbi.nlm.nih.gov/genbank/, the accession numbers were BankIt2624165 GnRHR1 OP482096, BankIt2624665 GnRHR2 OP482097, BankIt2622642 GnRHR3 OP433498, BankIt2622651 GnRHR4 OP433499, BankIt2622656 GnRH2 OP433500, BankIt2622662 GnRH3 OP433501, respectively.

## Ethics statement

The animal study was reviewed and approved by Huazhong Agricultural University.

## Author contributions

Data curation, CX and WL; formal analysis, YX and HZ; funding acquisition, GH and ZY; investigation, YO, RD, and YX; methodology, WL and RD; resources, XG; software, WL; supervision, GH; writing—original draft, WL and CX; writing—review and editing, GH. All authors contributed to the article and approved the submitted version.

## Funding

Funding support was provided by the National Key R&D Program of China (2018YFD0900404 to ZY), China Postdoctoral Science Foundation (2019M662747 to GH), and Hubei Province Postdoctoral Science Foundation (237934 to GH).

## Acknowledgments

This article is dedicated to Prof. Anderson O.L. Wong (University of Hong Kong, China) for his genuine interest in training young scientists in the field of comparative endocrinology.

## Conflict of interest

The authors declare that the research was conducted in the absence of any commercial or financial relationships that could be construed as a potential conflict of interest.

## Publisher’s note

All claims expressed in this article are solely those of the authors and do not necessarily represent those of their affiliated organizations, or those of the publisher, the editors and the reviewers. Any product that may be evaluated in this article, or claim that may be made by its manufacturer, is not guaranteed or endorsed by the publisher.

## Abbreviations

GnRH, gonadotropin-releasing hormone; LH, luteinizing hormone; FSH, follicle stimulating hormone; GnRHR, gonadotropin-releasing hormone receptor; GtHα, gonadotropic hormone alpha; INHBA, inhibin-βA; SgII, secretogranin II; POMCb, proopiomelanocortin B; CART2, cocaine and amphetamine-regulated transcript II; UTS1, urotensin 1; NMB, neuromedin-B; LEPR, leptin receptor; AC, adenylate cyclase; PKA, protein kinase A; PLC, phospholipase C; IP3, inositol trisphosphate PKC, protein kinase C; CaM, calmodulin; CaMK, calmodulin kinase; DRD2, dopamine D2 receptor; NFAT, nuclear factor of activated T cells; ORF, open reading frame; RT-PCR, real-time PCR; TMD, transmembrane domain.
