# Supplementary material for: Novel pituitary actions of GnRH in teleost: The link between reproduction and feeding regulation
Source: Front Endocrinol (Lausanne). 2022 Oct 11;13:982297. doi: 10.3389/fendo.2022.982297 (PMC9595134; doi:10.3389/fendo.2022.982297)
Supplement: Supplementary file 1 [file DataSheet_1.docx]

**SUPPLEMENTARY INFORMATION**

**Novel pituitary actions of GnRH in teleost: the link between reproduction and feeding regulation**

Wei Li^#^, Ruixin Du^#^, Chuanhui Xia^#^, Huiying Zhang, Yunyi Xie, Xiaowen Gao, Yu Ouyang, Zhan Yin*, Guangfu Hu*

^1^College of Fisheries, Hubei Province Engineering Laboratory for Pond Aquaculture, Huazhong Agricultural University, Wuhan, 430070, China

^2^State Key Laboratory of Freshwater Ecology and Biotechnology, Institute of Hydrobiology, Chinese Academy of Sciences, Wuhan 430072, China

**Supplementary Table S1**│**Signal pathway inhibitors used in *in vitro* experiments**

| name | function | Sales company | article number |
| --- | --- | --- | --- |
| H89 | PKA inhibitor | Calbiochem | 371963 |
| 2-APB | IP3 receptor retardant | Calbiochem | 100065 |
| KN62 | CaMK-II retardant | Calbiochem | 422706 |
| U-73122 | PLC inactivator | Sigma | U6756 |
| GF109203X | PKC inhibitor | Sigma | B6292 |
| Nifedipine | VSCC retardant | Sigma | N7634 |
| Calmidazolium | CaM antagonist | RBI | C-100 |
| MDL12330A | AC inhibitor | Merck | 444200 |

**Supplementary Table S2**│**Primer sequences and PCR conditions for ORF of GnRHRs in grass carp**

| Gene | Forward primer | Reverse primer | Annealing Tm (℃) | Product size (bp) |
| --- | --- | --- | --- | --- |
| *gnrhr1* | ATGTTAAAGATCTTTTGTC | CTATGCTTGTTGTCCACTG | 51 | 1157 |
| *gnrhr2* | ATGAACTCAACTCGTCACA | TTAGTCTATACAGGTGGAA | 50 | 1245 |
| *gnrhr3* | ATGTCTGATAACTCGTCCCT | TTACGTGGCCTTCGGCGGGT | 56 | 1077 |
| *gnrhr4* | ATGAATGACAGCTCTCCAAC | CTATTCCTTGTTTTTACTCT | 50 | 1218 |

**Supplemental Table S3**│**Primer sequences and PCR conditions for real-time PCR of GnRHs, GnRHRs and selected gene targets in grass carp**

|  | Forward primer | Reverse primer | Annealing Tm (℃) | Product size (bp) |
| --- | --- | --- | --- | --- |
| *gnrh2* | TGTGTCTAGGTGCCCAGTTTG | GCATCCAGCAGTATTGTCTTCA | 60 | 187 |
| *gnrh3* | ACTGGTCATACGGTTGGCTTC | CCTCGTCTGTTGGGAAATCTCT | 60 | 202 |
| *gnrhr1* | CACCGACATTCACCGTTGCT | CAGACTCACCACCACCAGGAT | 60 | 317 |
| *gnrhr2* | CGTGCTTTACTCAGCCAACC | CCTCCTCGCTTTGTTGATAG | 59 | 280 |
| *gnrhr3* | GCAGGAGACGGCGTACAACAT | CCAGCAGACCACGAACGACAT | 62 | 226 |
| *gnrhr4* | CAATAGCACAGGTGAAGCGG | CGCCAACCACTGAACCGTAA | 60 | 256 |
| *inhba* | GGTGCGACAGATGGACGAT | GAGTGGAAGGACAGCGAGTT | 55 | 225 |
| *sn2* | ACCAGAAAGCAACAGCAACGC | TAACTCGGACAGGAACAAGCC | 60 | 210 |
| *drd2* | ACTTAAACCCTCCGAGAC | TACACCAAGGACAATAGC | 58 | 296 |
| *lhβ* | ACATCCTCCTTCTCTTATTCTG | CAAGCGGACCGTCTCATAG | 58 | 204 |
| *gthα* | GATATGACTAACTTTGGATGTG | TAGTAACAGGTGCTACAGTGG | 58 | 227 |
| *fshβ* | TTCGTTGTTATGGTGATGCT | CGTGAAAACCGAGTCAGTCC | 60 | 198 |
| *pomcb* | TGAAGAAGAGGTTGAGGAAGAGAGT | GCTTGTCAGGTGGTGCGTTC | 60 | 217 |
| *cart2* | CGCTGCTGCTCTGCTTGT | GCGGACAATCGCACATCT | 60 | 201 |
| *uts1* | CTGACCACCCACATCCCCCT | AGTTCCGCCTGTTCCCTTTG | 60 | 195 |
| *nmba* | AAGAGTATTTCAAACAGCCAGC | GCTGACCAATTCTGTAAATAGC | 56 | 202 |
| *nmbb* | AGCCTTGACTTGACTGAACTAA | CGGAACTTTAGGGCGGAT | 60 | 249 |
| *lepr* | TGGAGCCAACCTTTCTATT | TTCTTGCTCTGGCAGGTAATA | 60 | 341 |
| *β-actin* | CTGGTATCGTGATGGACTCT | AGCTCATAGCTCTTCTCCAG | 56 | 285 |


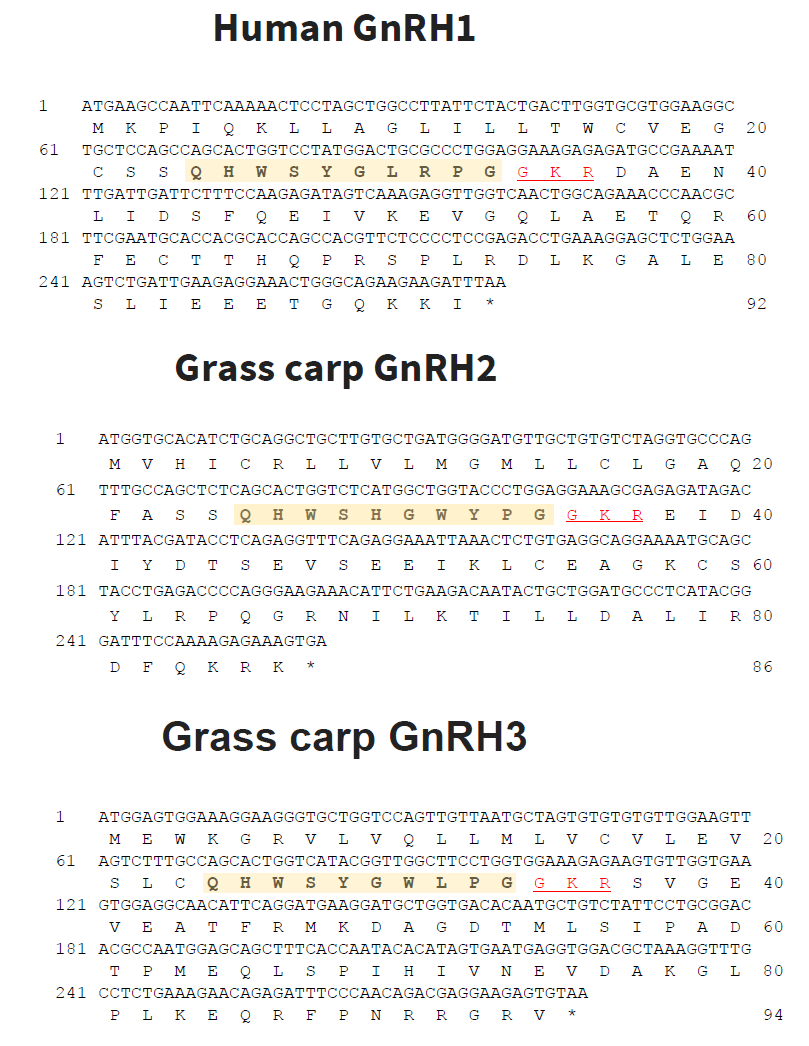


**Supplemental Figure S1**│**Molecular cloning of human Gnrh1, grass carp Gnrh2 & Gnrh3**. The open reading frame (ORF) of Gnrhs cDNA is shown in upper case letters. The stop codon is marked by an asterisk. Numbering of the deduced amino acid sequences begins with the first methionine of the ORF to the right of each line. Nucleotide numbers are the left of each line. In the corresponding protein sequence, the 10-amino acid Gnrh1 (QHWSYGLRPG), Gnrh2 (QHWSHGWYPG) and Gnrh3 (QHWSYGWLPG) is boxed in yellow. The cleavage sites (GRR) flanking the mature peptides are underlined for identification.


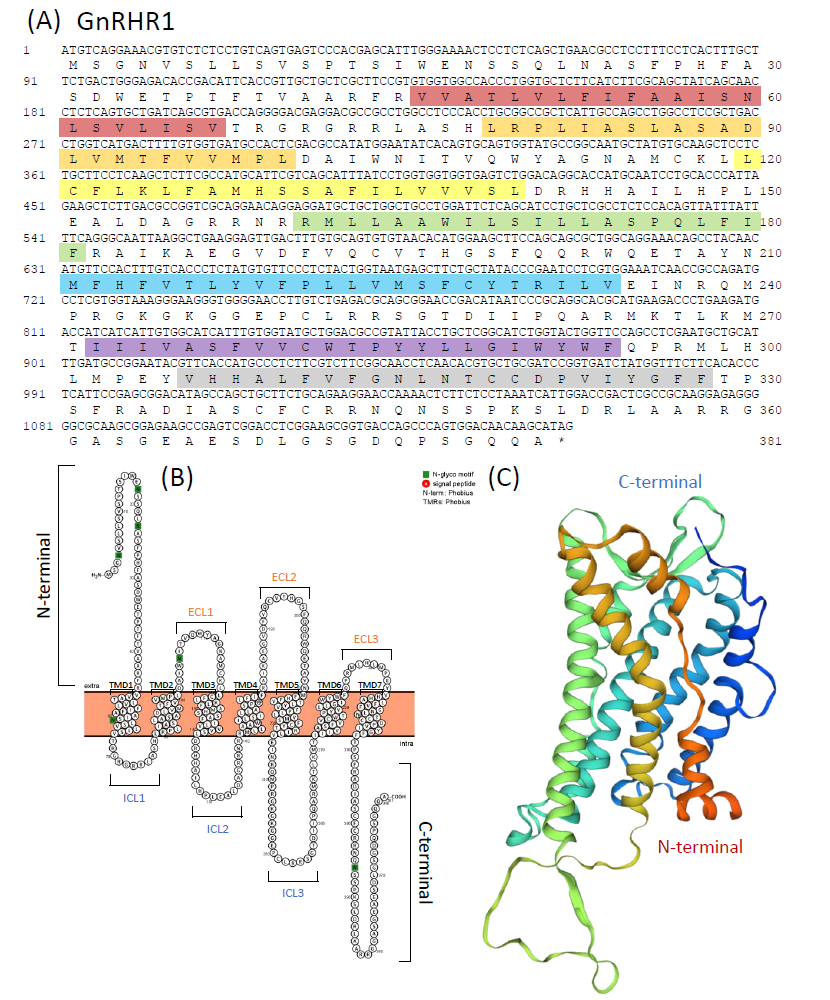


**Supplemental Figure S2**│**Molecular cloning and sequence alignment of grass carp Gnrhr1.** **(A)** Nucleotide and deduced amino acid sequences of grass carp Gnrhr1. Numbering of the deduced amino acid sequences begins with the first methionine of the ORF to the right of each line. Nucleotide numbers are to the left of each line. Predicted transmembrane domains (TMD1-7) are colored. **(B)** Snake diagram of grass carp Gnrhr1 with Protter program. The seven transmembrane domains, three intracellular domains and three extracellular domains are labeled as TMD1-7, ICL1-3 and ECL1-3, respectively. Sequence identities of transmembrane domains and extracellular domains, intracellular domains, N-termini and C-termini between grass carp and other vertebrates were showed in the Table. **(C)** 3-D protein model of grass carp Gnrhr1 was deduced based on the crystal structure of human Gnrhr using SWISS-MODEL program. The location of N-terminal and C-terminal were highlighted, respectively. The amino acids with hydrophobic side chains are colored blue, while those with hydrophilic side chains are colored red.


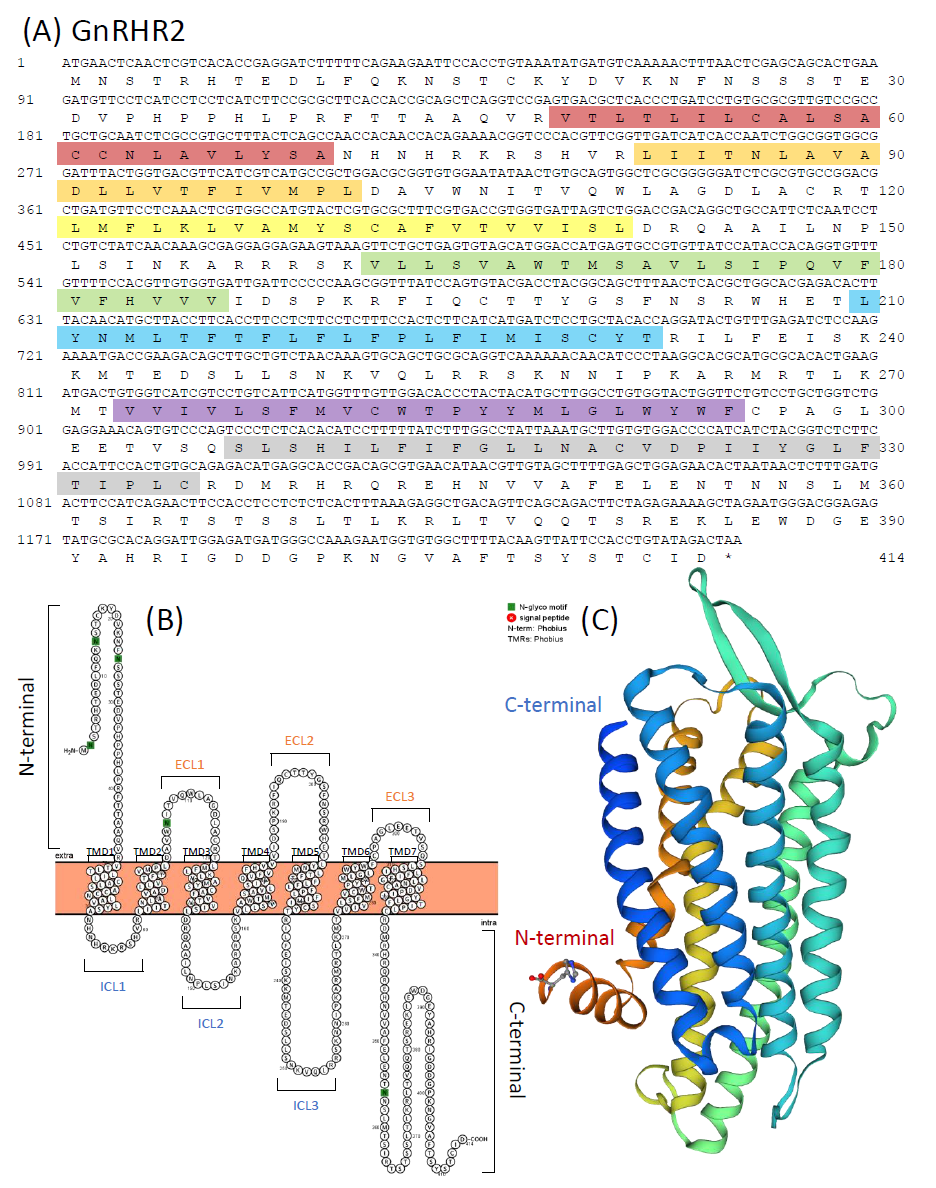


**Supplemental Figure S3**│**Molecular cloning and sequence alignment of grass carp Gnrhr2.** **(A)** Nucleotide and deduced amino acid sequences of grass carp Gnrhr2. Numbering of the deduced amino acid sequences begins with the first methionine of the ORF to the right of each line. Nucleotide numbers are to the left of each line. Predicted transmembrane domains (TMD1-7) are colored. **(B)** Snake diagram of grass carp Gnrhr2 with Protter program. The seven transmembrane domains, three intracellular domains and three extracellular domains are labeled as TMD1-7, ICL1-3 and ECL1-3, respectively. Sequence identities of transmembrane domains and extracellular domains, intracellular domains, N-termini and C-termini between grass carp and other vertebrates were showed in the Table. **(C)** 3-D protein model of grass carp Gnrhr2 was deduced based on the crystal structure of human Gnrhr using SWISS-MODEL program. The location of N-terminal and C-terminal were highlighted, respectively. The amino acids with hydrophobic side chains are colored blue, while those with hydrophilic side chains are colored red.


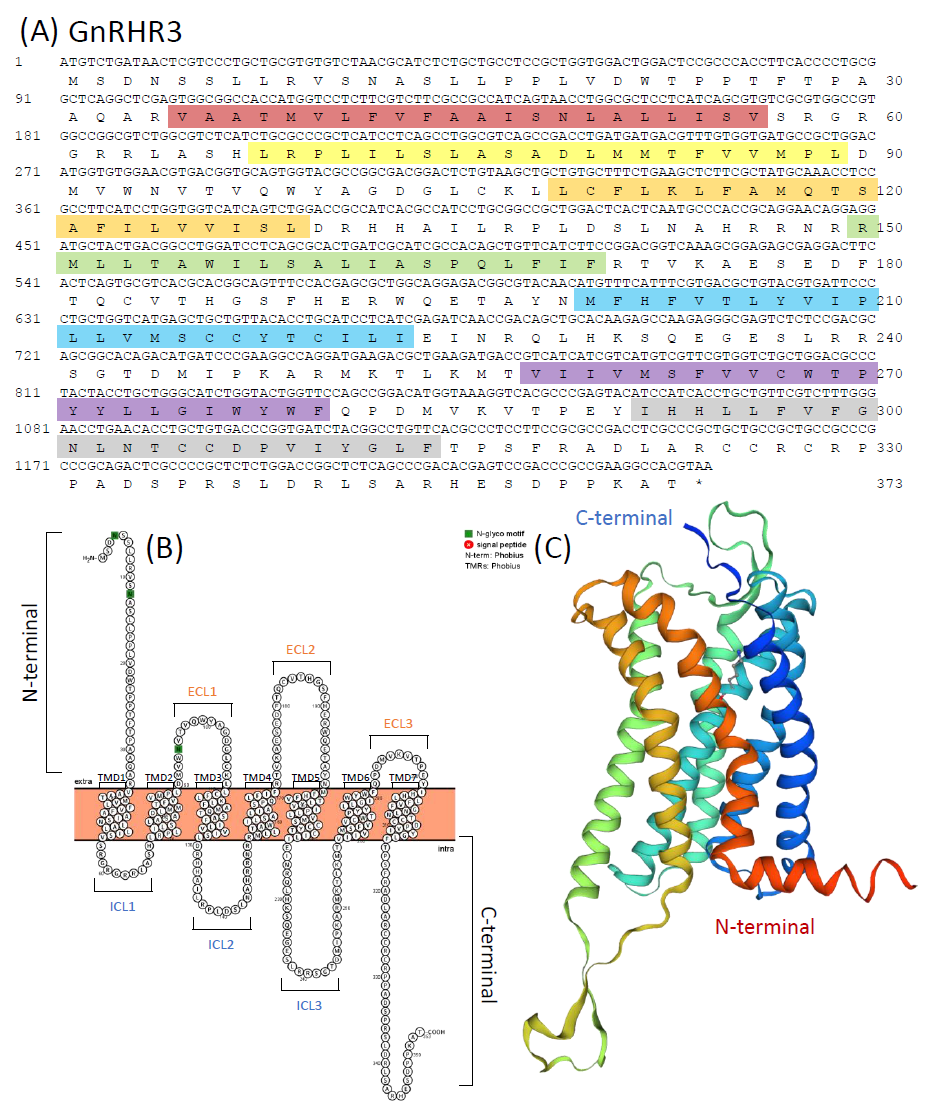


**Supplemental Figure S4**│**Molecular cloning and sequence alignment of grass carp Gnrhr3. (A)** Nucleotide and deduced amino acid sequences of grass carp Gnrhr3. Numbering of the deduced amino acid sequences begins with the first methionine of the ORF to the right of each line. Nucleotide numbers are to the left of each line. Predicted transmembrane domains (TMD1-7) are colored. **(B)** Snake diagram of grass carp Gnrhr3 with Protter program. The seven transmembrane domains, three intracellular domains and three extracellular domains are labeled as TMD1-7, ICL1-3 and ECL1-3, respectively. Sequence identities of transmembrane domains and extracellular domains, intracellular domains, N-termini and C-termini between grass carp and other vertebrates were showed in the Table. **(C)** 3-D protein model of grass carp Gnrhr3 was deduced based on the crystal structure of human Gnrhr using SWISS-MODEL program. The location of N-terminal and C-terminal were highlighted, respectively. The amino acids with hydrophobic side chains are colored blue, while those with hydrophilic side chains are colored red.


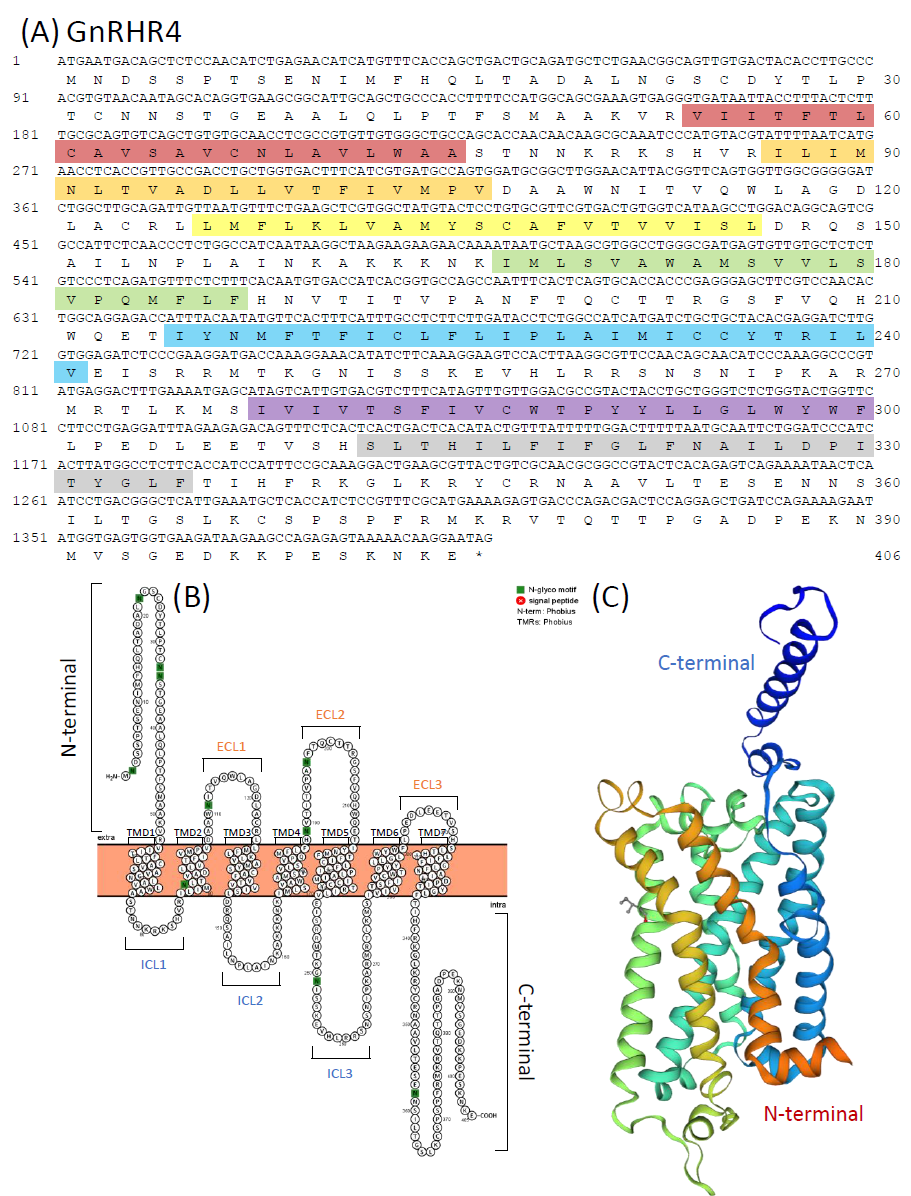


**Supplemental Figure S5**│**Molecular cloning and sequence alignment of grass carp Gnrhr4.** **(A)** Nucleotide and deduced amino acid sequences of grass carp Gnrhr4. Numbering of the deduced amino acid sequences begins with the first methionine of the ORF to the right of each line. Nucleotide numbers are to the left of each line. Predicted transmembrane domains (TMD1-7) are colored. **(B)** Snake diagram of grass carp Gnrhr4 with Protter program. The seven transmembrane domains, three intracellular domains and three extracellular domains are labeled as TMD1-7, ICL1-3 and ECL1-3, respectively. Sequence identities of transmembrane domains and extracellular domains, intracellular domains, N-termini and C-termini between grass carp and other vertebrates were showed in the Table. **(C)** 3-D protein model of grass carp Gnrhr4 was deduced based on the crystal structure of human Gnrhr using SWISS-MODEL program. The location of N-terminal and C-terminal were highlighted, respectively. The amino acids with hydrophobic side chains are colored blue, while those with hydrophilic side chains are colored red.


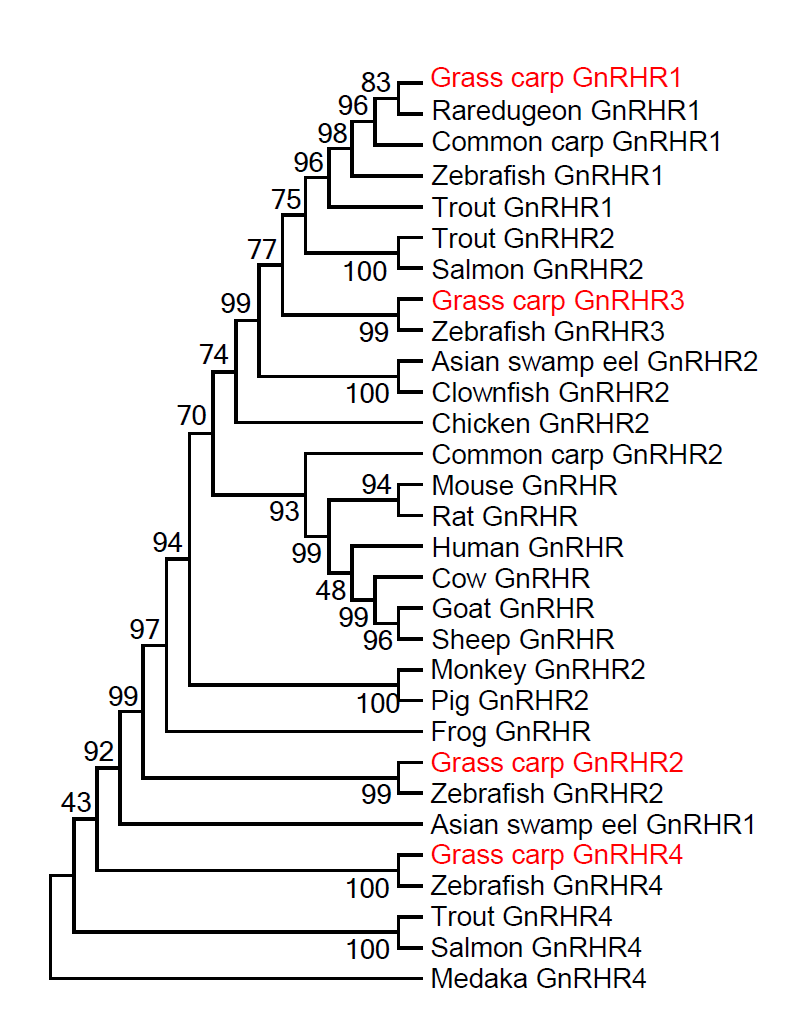


**Supplemental Figure S6**│**Phylogenetic analysis of vertebrate Gnrh amino acid sequences using neighbor-joining method with MEGA X.** The grass carp Gnrhr1, Gnrhr2, Gnrhr3 and Gnrhr4 were obtained and used to cluster into four branches. The numbers presented in the guide tree are the percentage of bootstrap values based on 1000 bootstrap. The other Gnrhrs were collected form NCBI database.


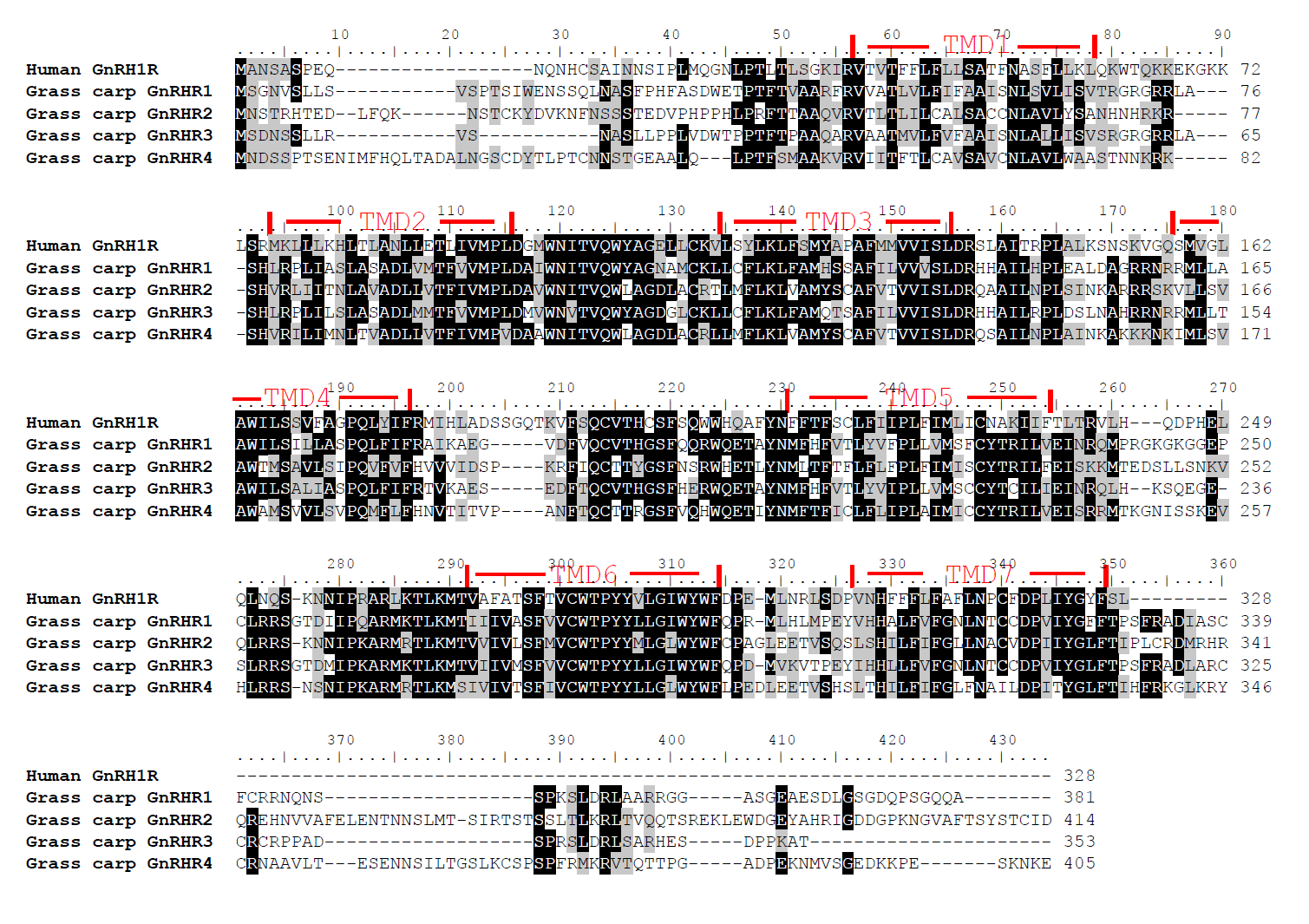


**Supplemental Figure S7**│**Sequence alignment of grass carp Gnrhr1, Gnrhr2, Gnrhr3 and Gnrhr4 and human Gnrhr.** Protein sequence alignment of grass carp Gnrhr1, Gnrhr2, Gnrhr3 and Gnrhr4 and human Gnrhr using Clustal-W algorithm with MacVector program. The lower conserved a.a. residues are boxed in grey where higher are in black. The seven transmembrane domainsare labeled as TMD1-7, respectively.
